# Three-dimensional continuous gait trajectory estimation using single Shank-Worn inertial measurement units and clinical walk test application

**DOI:** 10.1038/s41598-022-09372-w

**Published:** 2022-03-30

**Authors:** Hirotaka Uchitomi, Yuki Hirobe, Yoshihiro Miyake

**Affiliations:** Department of Computer Science, School of Computing, Tokyo Institute of Technology, Yokohama, 226-8502 Japan

**Keywords:** Physical examination, Rehabilitation, Information technology, Software

## Abstract

State-of-the-art estimation methods using inertial measurement units (IMUs) for global continuous gait path and local stepwise gait trajectory during walking have been developed. However, estimation methods for continuous gait trajectory integrating both these aspects with high accuracy are almost lacking. Thus, continuous gait trajectory estimation using a single shank-worn IMU with high accuracy is proposed in this study. This method calculates three-dimensional local stepwise gait trajectory based on IMU measurement data extracted between adjacent middle points of stance phases during walking. Continuous gait trajectory is estimated by concatenating adjacent local stepwise gait trajectories based on relative angles determined according to stride vectors and shank orientations. Evaluation experiments results obtained using the optical motion capture system with 12 healthy participants demonstrated estimation errors in the stride length (− 0.027 (− 0.054 to − 0.006) m) and turning angle (0.7 (− 0.2–1.7)°), and normalized endpoint position error (0.029 (0.019–0.04) m). Comparing with previous reports, the proposed method integrally achieves a continuous gait trajectory with a low estimation error level in both local and global aspects despite the continuous measurement of multiple gait cycles. The proposed simple and low-cost method can be applied in the medical field and contribute to expansion of the application of precise gait information in daily life.

## Introduction

There has been accelerated research and development of simple and low-cost inertial measurement units (IMUs) that can be used to accurately estimate position information for the measurement and analysis of gait^1,2^. Methods such as the estimation method of global continuous gait path^3–6^, which provides the continuous polyline based on connecting adjacent foot ground contact points during walking in the whole as global tracking gait information, and the estimation method of local stepwise gait trajectory^2,7–13^, which provides the stepwise three-dimensional (3D) detailed position time series during walking in each gait cycle for step-by-step as local precise gait information, have been implemented previously. It is expected that gait measurement using an IMU will be applied to pedestrian dead reckoning (PDR) for human activity tracking^14^ and clinical gait analysis in evidence-based medicine and therapy in the diagnosis^5,13,15^. In fact, as a example of such functional gait assessment, the gait assessment of the foot based on Performance Oriented Mobility Assessment (POMA) evaluates not only the length and height of the stride and the symmetry of the strides on the local stepwise gait trajectory, but also the path straightness on the global continuous gait path^16,17^.

Step-and-heading system (SHS) is one of the most representative methods^2^ among the previous studies on the estimation methods for the global continuous gait path measurement^1^. The SHS method estimates the stride vector by dividing the raw data measured by the IMU while walking into each gait cycle and performing integration processing for each of the divided data, and then, by concatenating these stride vectors to estimate the global continuous gait path. Previous studies attached IMU sensor on the shoes and attempted to track long-distance closed gait paths^3,4^ and estimate some medium-distance gait paths^6^. However, the moving artifacts of the footwear and also the soft tissue of the lower shank affect the estimation error^18^. Other study^5^ used the shank-worn IMU and estimated the trajectory using conventional zero velocity update (ZUPT) and simple integration of angular velocity^19^. However, the drift of the measurement data may not always be linear, and variety of the drifts and artifacts may also affect the estimation accuracy. Additionally, these studies were limited to the estimation of global continuous gait paths that linearly connect adjacent foot ground positions and do not extend to implementing the estimation of the local stepwise gait trajectory of precise foot motion between adjacent foot ground positions.

The improvement in the estimation accuracy was investigated by processing the raw data measured during walking for estimating the local stepwise gait trajectory. The raw data were then divided into a gait cycle because the velocity of the IMU attached to the foot during the stance phase of walking is near zero owing to the timing of foot ground contact and foot stop^2,7,8,13,20,21^. Furthermore, methods have been proposed to improve accuracy by applying foot motion modeling such as modeling a two-dimensional (2D) inverted pendulum, where the IMU motion is considered a circular motion in the sagittal plane^9,22^, a 3D inverted pendulum^10^, and model fitting approach^11^. This previous study using the model fitting approach eliminated the drift and artifact using parabolic model to extract the heading and combination of bi-directional integration of measurement data. However, these previous studies are limited to estimating the local stepwise gait trajectory and not implementing the global continuous gait path estimation, and also directional changes between adjacent strides were not taken into account. Previous study^13^ estimating the turning angle of foot in each gait cycle was also basically just an application of simple integration.

Based on these previous studies, the global continuous gait path during walking and local stepwise gait trajectory during walking have been investigated; however, there is almost no implementation of an estimation method for continuous gait trajectory that integrates both aspects, which remains a problem. In shank worn IMU based SHS, estimation method for directional changes between adjacent strides is considered to have room for improvement. In view of these remaining problems, this study aims to construct and propose a new method for estimating the continuous gait trajectory with high accuracy. To this end, the current study was conducted with the aim of integrating both the estimation of global continuous gait path and local stepwise gait trajectory. The proposed method was extended to the estimation of continuous gait trajectory by continuously connecting adjacent local stepwise gait trajectories using a single shank-worn IMU based on the authors’ previous outcome^11^. The proposed method calculates the middles of stance phases during walking using IMU measurement raw data and local stepwise gait trajectory in three dimensions. The continuous gait trajectory is estimated by concatenating adjacent local stepwise gait trajectories based on the relationship of the relative angle, which is determined based on stride vectors and shank orientations (Table 1).

**Table 1:** The characteristics of the experimental participants including the number, the age, the tall, the weigh, and the shoe size in the median (1st quartile–3rd quartile), respectively.

Participant	Number [#]	Age [Year]	Tall [cm]	Wight [kg]	Shoe size [cm]
All	12	23 (23–24.5)	170 (165–176.5)	62 (55.25–66.25)	26 (25.25–26.75)
Male	9	24 (23–26)	172 (170–178)	65 (60.5–70)	26.5 (26–27.5)
Female	3	23 (22.5–23)	161 (158–163)	49 (47–55.5)	24 (23.5–24.25)

## Results

**Figure 1:**
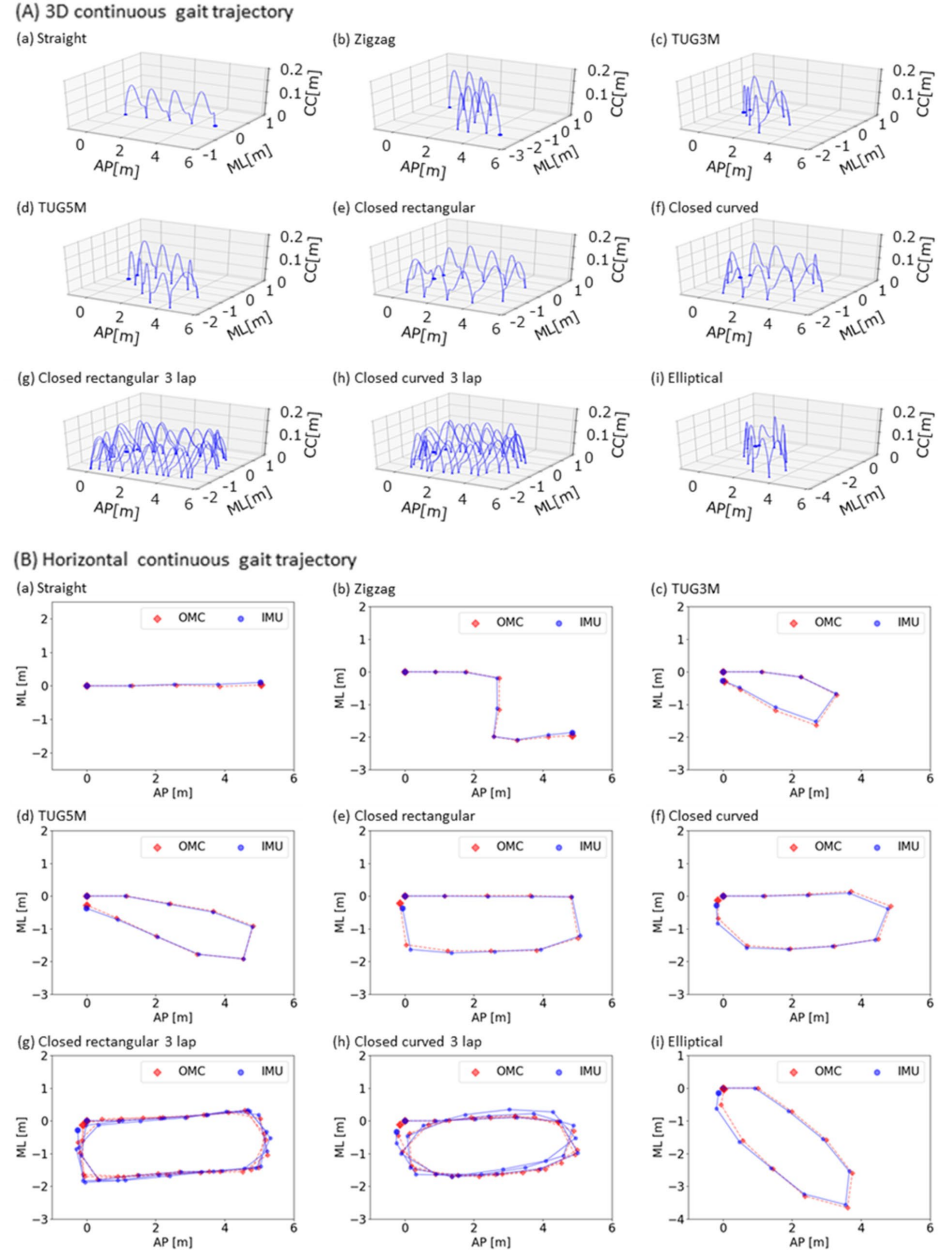
Examples of continuous gait trajectory estimated by the proposed method with single shank-worn IMU in the nine walking route conditions. (**A**) 3D continuous gait trajectory, (**B**) Horizontal continuous gait trajectory, (**a**) straight, (**b**) zigzag, (**c**) TUG3M, (**d**) TUG5M, (**e**) closed rectangular, (**f**) closed curved, (**g**) closed rectangular 3 lap, (**h**) closed curved 3 lap, and (**i**) elliptical. In the axes, CC means craniocaudal direction, AP means anterior–posterior direction, and ML means medio-lateral direction.

In the evaluation experiment for the proposed method, nine males and three females, a total of 12 participants participated as the experimental participants in the current study. Figure 1(A) shows examples of continuous gait trajectories estimated by the proposed method under nine walking route conditions. The global continuous gait path and local stepwise gait trajectory can be simultaneously visualized in the resultant examples. Figure 1(B) shows examples of horizontal continuous gait trajectory for the nine walking route conditions. Owing to accuracy evaluation, the gait trajectory acquired by optical motion capture system (OMC) as the gold standard is displayed in overlapping. The results of the proposed method and the gold standard overlap. In the evaluation experiment, the proposed method was evaluated from two aspects: the segmental evaluation and the entire evaluation. The segmental evaluation verified the accuracy of stride length and turning angle. The entire evaluation verified the accuracy of the walking endpoint. Table 2 shows the data number, the trial duration and the walk speed of the experimental results in each condition based on the route conditions and the speed conditions, respectively. Here, the statistical results are basically provided using median (1st quartile–3rd quartile) in the following reports, where the output data in evaluations was for non-normal data because of the results from Shapiro–Wilk test.

**Table 2:** Data number, the trial duration and the walk speed of the experimental results in each condition based on the route conditions and the speed conditions in median (1st quartile–3rd quartile).

Route condition	Speed condition	Data Number [#]	Trial duration [s]	Walk speed [m/s]
Straight	Normal speed	5 (5–5)	6.69 (6.32–7.13)	0.81 (0.56–0.98)
	Slow speed	6 (5–6)	9.2 (8.34–10.83)	0.56 (0.44–0.68)
Zigzag	Normal speed	7 (6–7)	8.7 (7.94–10.31)	0.73 (0.59–0.83)
	Slow speed	8 (7–8)	12.39 (11.03–14.23)	0.51 (0.42–0.61)
TUG3M	Normal speed	7 (7–7)	9.56 (8.42–9.82)	0.82 (0.72–0.97)
	Slow speed	8 (8–9)	13.7 (12.3–16.16)	0.53 (0.43–0.63)
TUG5m	Normal speed	10 (9–10)	12.28 (10.9–14.22)	0.9 (0.75–1.01)
	Slow speed	11.5 (11–12)	19.58 (16.21–21.71)	0.57 (0.47–0.69)
Closed rectangular	Normal speed	11.5 (11–12.25)	14.86 (13.88–16.12)	0.85 (0.72–0.99)
	Slow speed	13.5 (13–14.25)	22.3 (19.82–27.3)	0.57 (0.46–0.67)
Closed curved	Normal speed	10 (10–11)	13.55 (11.54–14.3)	0.87 (0.73–0.99)
	Slow speed	13 (11–13)	21.65 (17.86–23.18)	0.56 (0.48–0.66)
Closed rectangular 3 lap	Normal speed	33 (32–36)	41.86 (37.88–45.37)	0.91 (0.8–1.05)
	Slow speed	39.5 (36–41.25)	62.71 (55.1–70.29)	0.61 (0.52–0.71)
Closed curved 3 lap	Normal speed	29 (28–30.5)	37.34 (33.21–39.98)	0.9 (0.8–1.06)
	Slow speed	35 (32–37)	55.15 (48.33–65.7)	0.61 (0.49–0.73)
Elliptical	Normal speed	10 (10–11)	12.78 (12.33–15.71)	0.87 (0.72–1.01)
	Slow speed	13 (11.75–13)	20.9 (19.33–25.14)	0.56 (0.47–0.66)

**Figure 2:**
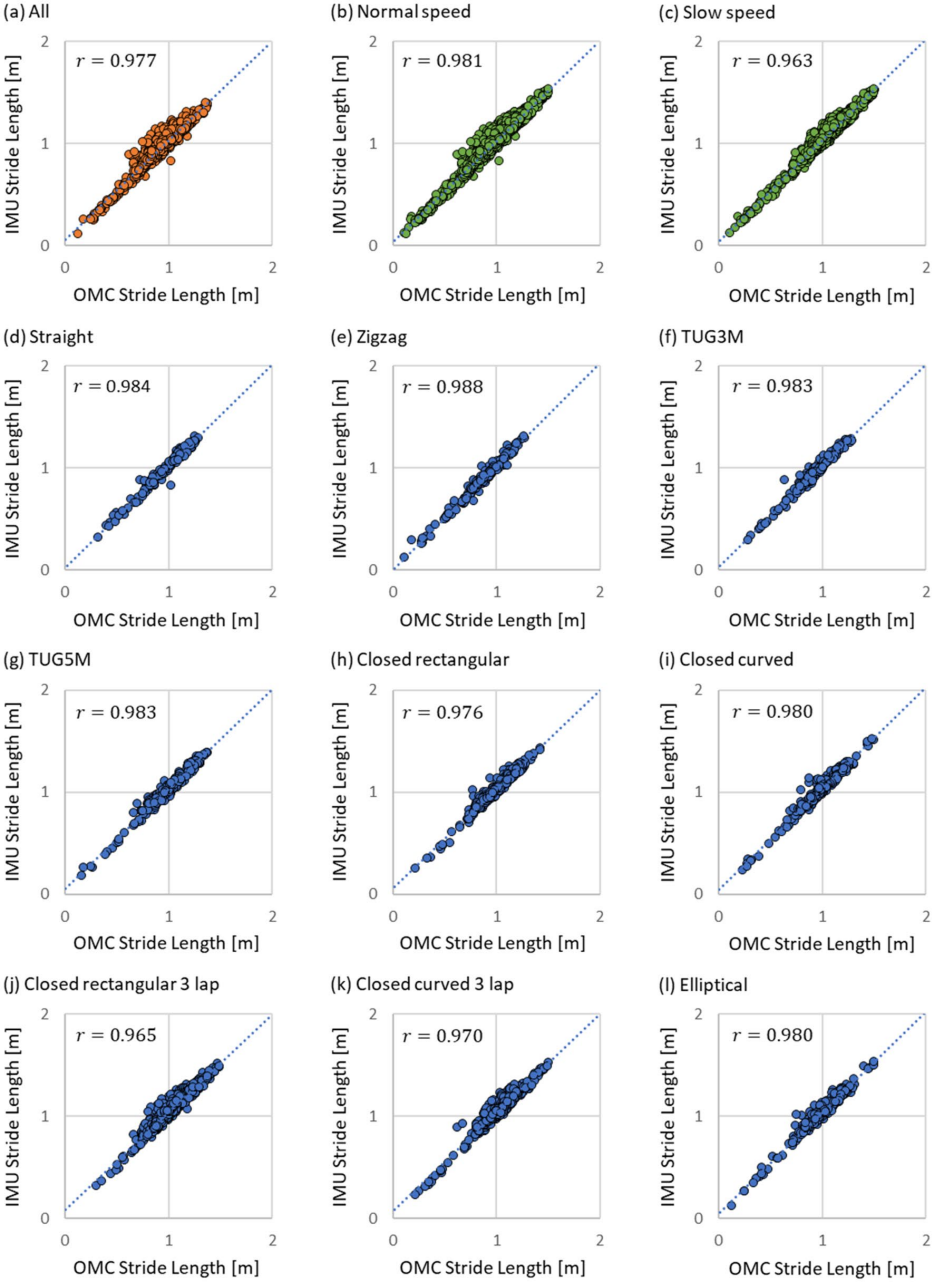
Results of Pearson's product ratio correlation analysis between the proposed method and golden standard in stride length. These results were evaluated for the two walking speed conditions and nine walking route conditions. IMU means the proposed method and OMC means the golden standard as the ground truth.

**Figure 3:**
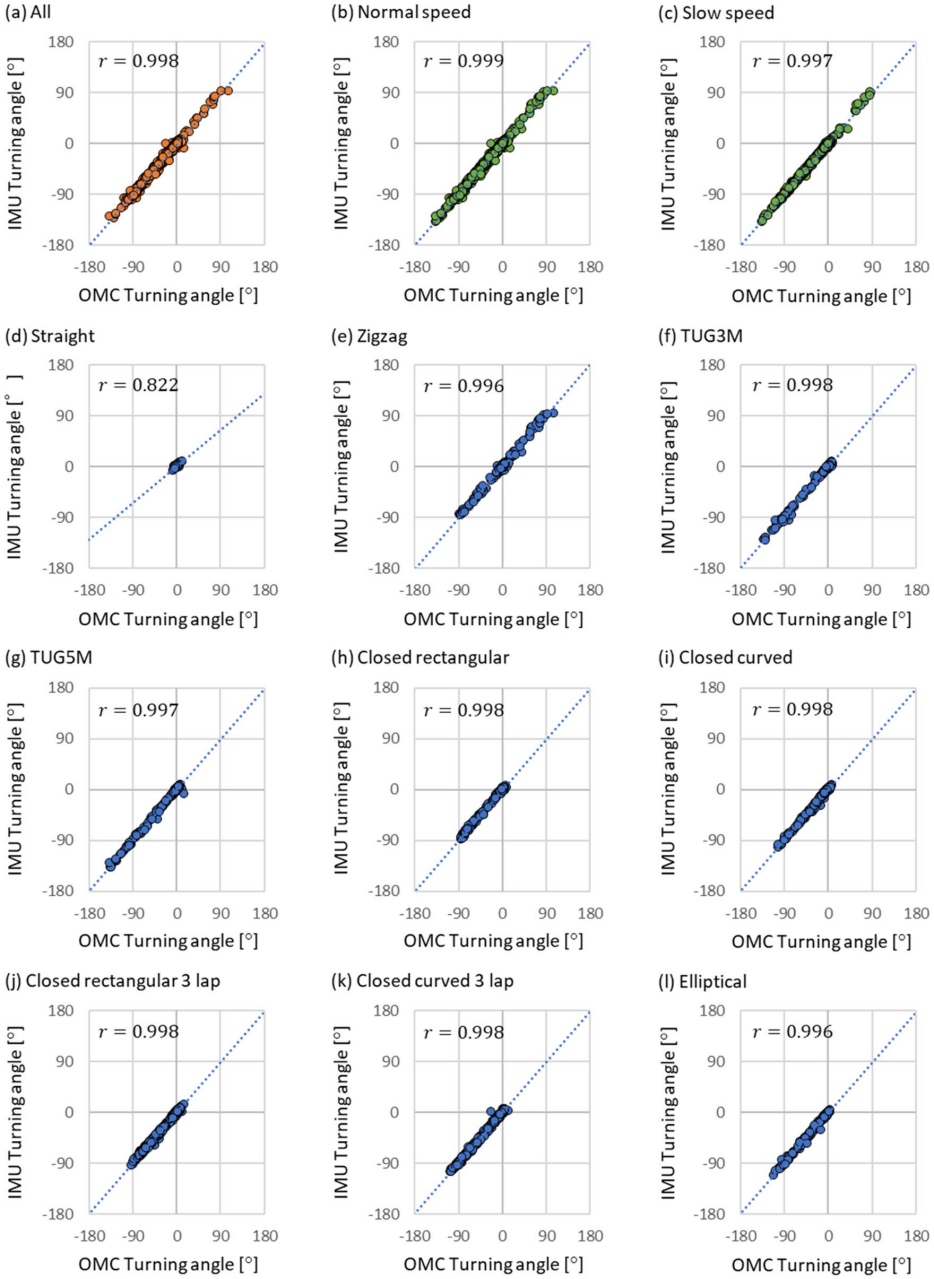
Results of Pearson's product ratio correlation analysis between the proposed method and the golden standard in turning angle. These results were evaluated for the two walking speed conditions and nine walking route conditions. IMU means the proposed method and OMC means the golden standard as the ground truth.

In the results of the segmental evaluation, Figs. 2 (a) and 3 (a) show the results of Pearson's product ratio correlation analysis between the proposed method and the golden standard in stride length and the turning angle in all experimental trials, respectively. The Pearson's product rate correlation coefficient R of the stride length was 0.977 with a p-value of less than 0.001. The R of the turning angle was 0.998 with a p-value of less than 0.001. Figure 2 (b) and (c) show the results of Pearson's product rate correlation analysis for two walking speed conditions in the stride length. Figure 2 (d)–(l) show the results of the analysis for the nine walking route conditions in the stride length. Figure 3 (b) and (c) show the results of the analysis for the two walking speed conditions in the turning angle. Figure 3 (d)–(l) show the results of the analysis for the nine walking route conditions in the turning angle. Table 3 summarizes Pearson's product rate correlation coefficients R and the p-value level for the correlation analysis results of the stride length shown in Figure 2 and correlation analysis results of the turning angle shown in Fig. 3.

**Table 3:** Pearson's product rate correlation coefficients R and p-value level between the proposed method and the golden standard in stride length and in the turning angle.

Experimental condition	Stride length [m]		Turning angle [°]	
	R	*P*-value	R	*P*-value
All	0.977	***	0.998	***
Normal speed	0.981	***	0.999	***
Slow speed	0.963	***	0.997	***
Straight	0.984	***	0.822	***
Zigzag	0.988	***	0.996	***
TUG3M	0.983	***	0.998	***
TUG5m	0.983	***	0.997	***
Closed rectangular	0.976	***	0.998	***
Closed curved	0.980	***	0.998	***
Closed rectangular 3 lap	0.965	***	0.998	***
Closed curved 3 lap	0.970	***	0.998	***
Elliptical	0.980	***	0.996	***

These results are evaluated under the two walking speed conditions and the nine walking route conditions. ***P < 0.001.

**Figure 4:**
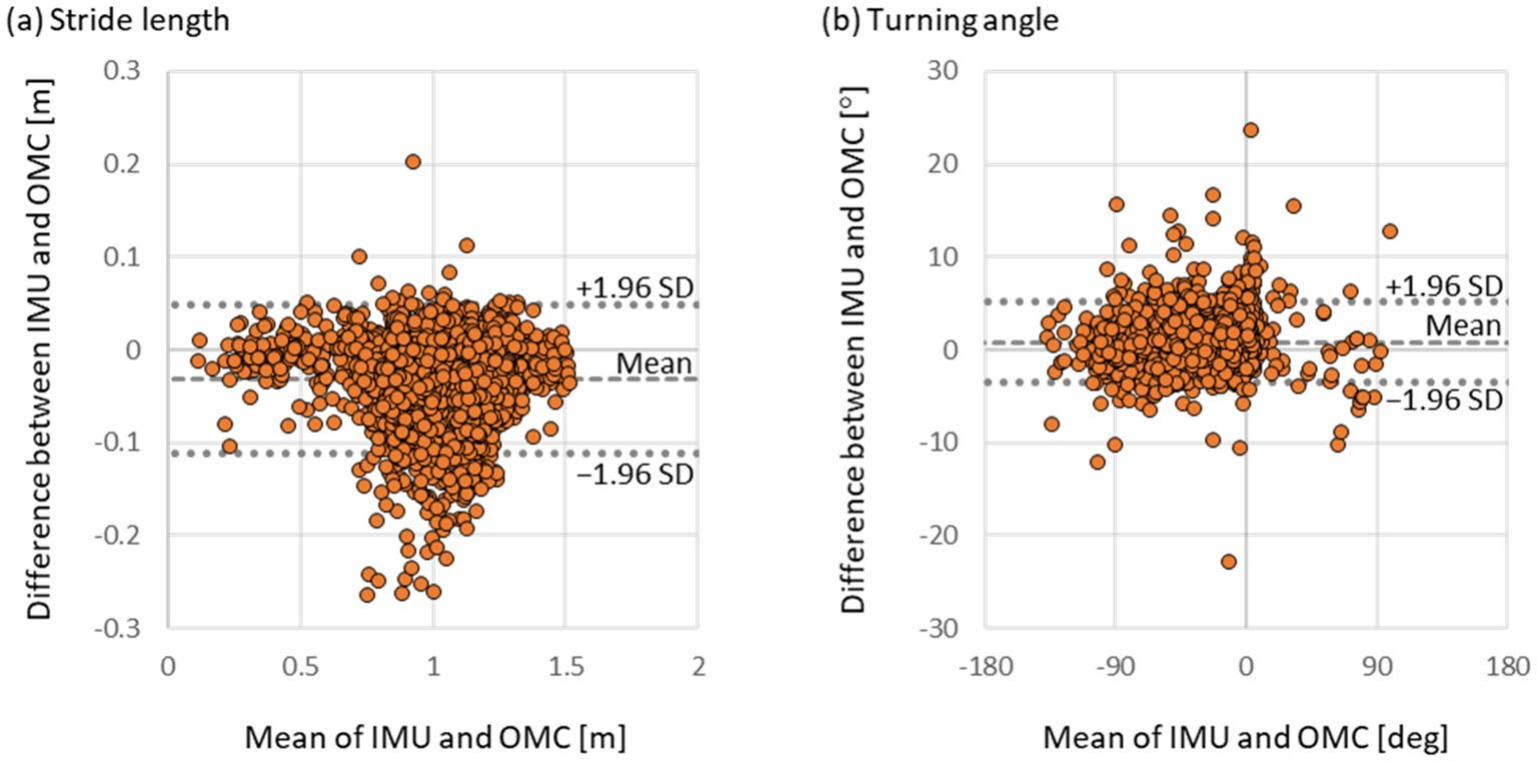
Results of the Bland–Altman analysis and the limits of agreement for stride length and turning angle in all experimental trials conducted under all conditions. IMU means the proposed method and OMC means the golden standard as the ground truth.

Figure 4(a) and (b) show the Bland–Altman analysis results for the stride length and turning angle for all experimental trials, respectively. The agreement between the results of the proposed method and the gold standard was verified by the limits of agreement (LOA) of the Bland–Altman analysis. The area of the LOA for the stride length includes 95.5% of the evaluated values in the analysis, which is greater than 95%. The area of the LOA for the turning angle includes 95.2% of the evaluated values in the analysis, which is greater than 95%.

**Figure 5:**
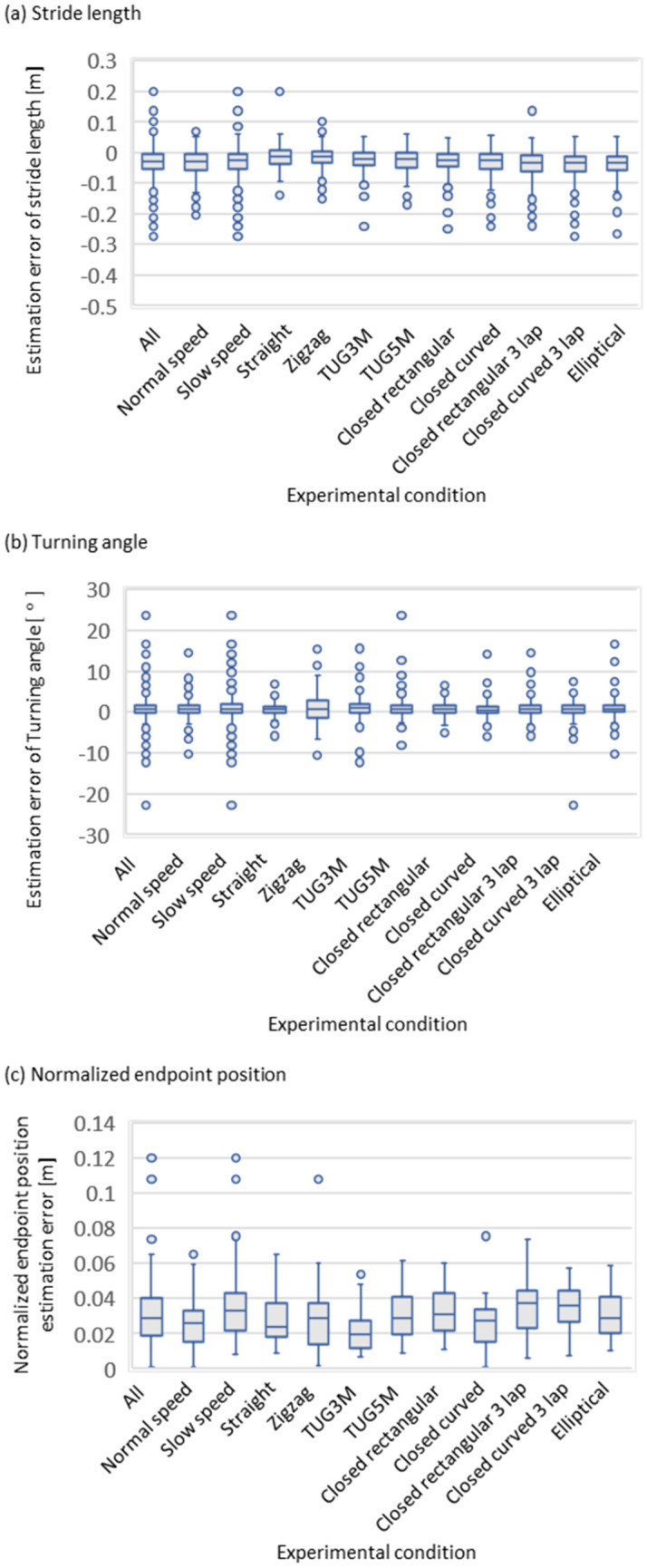
Box plot results of evaluating the estimation error of the proposed method compared to the golden standard in the stride length the turning angle, and the normalized endpoint position, respectively. These results are evaluated under two walking speed conditions and the nine walking route conditions.

Figure 5(a) and (b) show the results of evaluating the estimation error of the proposed method compared to the golden standard in stride length and turning angle, respectively.

In the results of the entire evaluation, the results of the normalized endpoint estimation error of the proposed method compared to the golden standard, where the estimation error was normalized by the number of gait cycles in the results, for the entire evaluation are summarized in Fig. 5(c). Table 4 summarizes the results regarding Fig. 5.

**Table 4:** Evaluation results of the estimation error of the proposed method compared to the golden standard in the stride length the turning angle, and the normalized endpoint position, respectively.

Condition	Stride length [m]	Turning angle [°]	Normalized endpoint position [m]
All	− 0.027 (− 0.054 to − 0.006)	0.7 (− 0.2–1.7)	0.029 (0.019–0.04)
Normal speed	− 0.028 (− 0.057 to − 0.006)	0.7 (− 0.2–1.5)	0.026 (0.016–0.033)
Slow speed	− 0.026 (− 0.051 to − 0.005)	0.9 (− 0.2–2)	0.033 (0.022–0.043)
Straight	− 0.011 (− 0.036 to 0.007)	0.6 (0–1.4)	0.024 (0.018–0.036)
Zigzag	− 0.012 (− 0.033 to 0.005)	0.7 (− 1.3–3)	0.029 (0.016–0.036)
TUG3M	− 0.022 (− 0.04 to − 0.001)	1.0 (− 0.2–2)	0.019 (0.013–0.025)
TUG5m	− 0.021 (− 0.049 to 0.001)	0.7 (− 0.2–1.7)	0.029 (0.02–0.039)
Closed rectangular	− 0.024 (− 0.046 to − 0.003)	0.8 (− 0.3–1.7)	0.031 (0.023–0.043)
Closed curved	− 0.026 (− 0.054 to − 0.007)	0.6 (− 0.3–1.4)	0.028 (0.016–0.031)
Closed rectangular 3 lap	− 0.032 (− 0.06 to − 0.008)	0.7 (− 0.2–1.7)	0.038 (0.024–0.044)
Closed curved 3 lap	− 0.033 (− 0.06 to − 0.012)	0.8 (− 0.1–1.8)	0.036 (0.027–0.044)
Elliptical	− 0.032 (− 0.059 to − 0.013)	0.8 (0.0–1.8)	0.029 (0.021–0.041)

These results are evaluated under the two walking speed conditions and nine walking route conditions.

**Figure 6:**
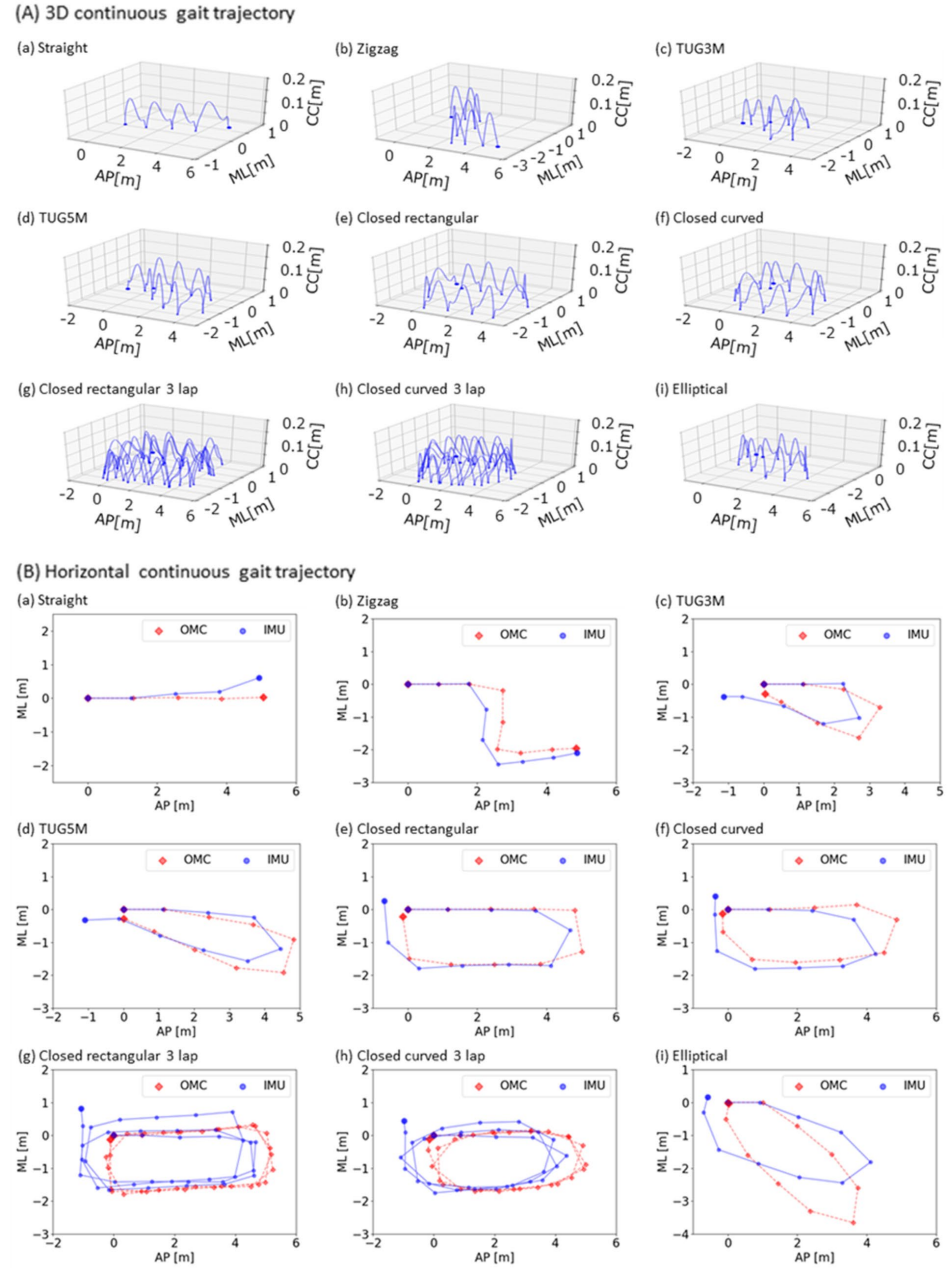
Examples of continuous gait trajectory estimated by the representative previous method with single shank-worn IMU in the nine walking route conditions. (**A**) 3D continuous gait trajectory, (**B**) Horizontal continuous gait trajectory, (**a**) straight, (**b**) zigzag, (**c**) TUG3M, (**d**) TUG5M, (**e**) closed rectangular, (**f**) closed curved, (**g**) closed rectangular 3 lap, (**h**) closed curved 3 lap, and (**i**) elliptical. In the axes, CC means craniocaudal direction, AP means anterior–posterior direction, and ML means medio-lateral direction.

**Figure 7:**
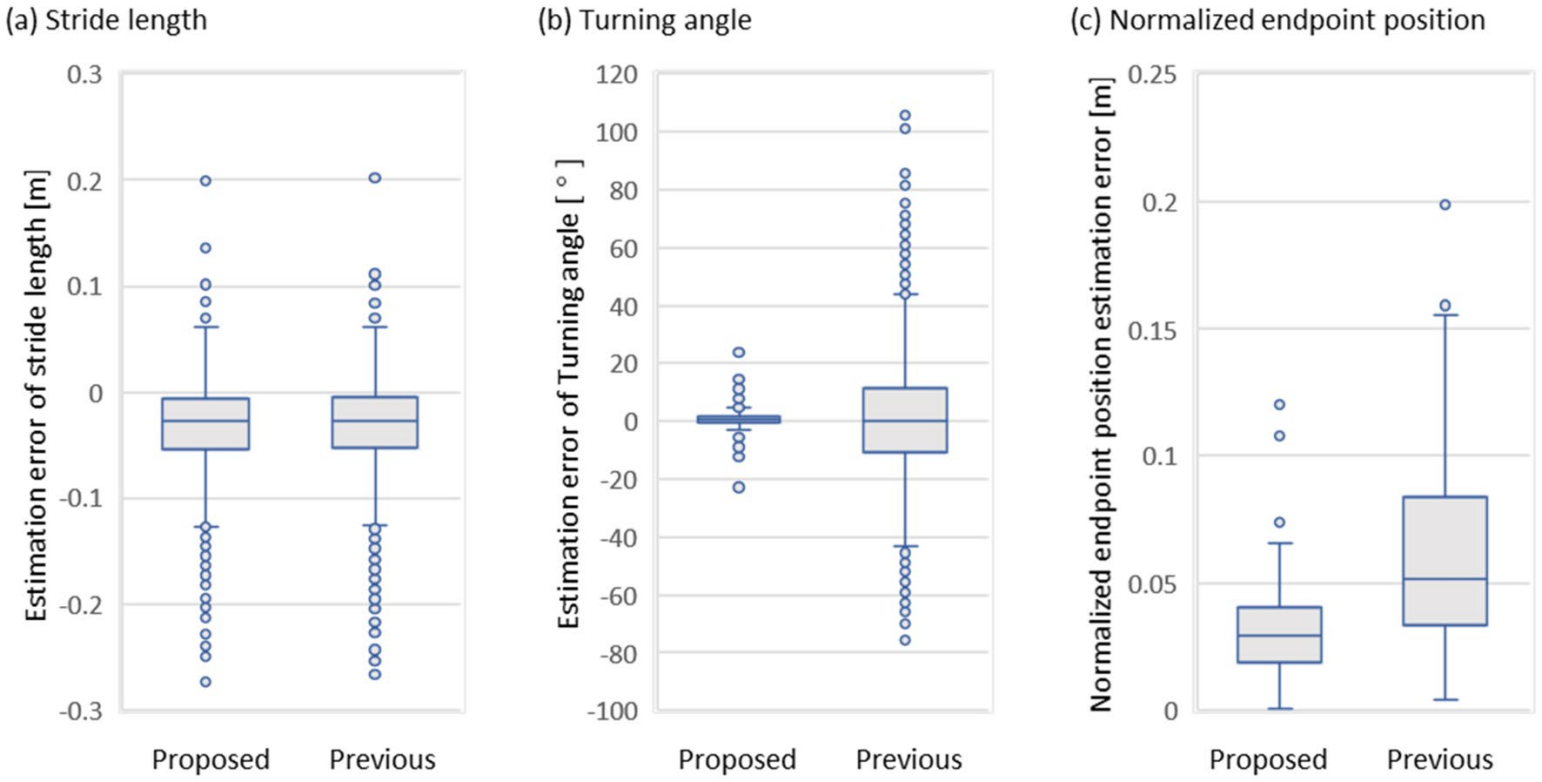
Box plot results in comparison of estimation error of stride length, estimation error of turning angle, and normalized endpoint position estimation error, between the proposed method and the representative previous method.

Moreover, in order to compare the proposed method to the previous method, the same IMU measured raw data was used in the previous method and the trajectories were estimated based on the previous method. Figure 6 shows the examples of continuous gait trajectory estimated by the previous method with single shank-worn IMU in the nine walking route conditions. Figure 7 and Table 5 shows the comparison results between the proposed method and the previous method.

**Table 5:** Comparison results of the estimation error between the proposed method and the previous method in the stride length the turning angle, and the normalized endpoint position, respectively.

Method	Stride length [m]	Turning angle [°]	Normalized endpoint position [m]
Proposed	− 0.027 (− 0.054 to − 0.006)	0.7 (− 0.2 to 1.7)	0.029 (0.019 to 0.040)
Previous	− 0.027 (− 0.053 to − 0.004)	0.3 (− 10.5 to 11.2)	0.052 (0.034 to 0.083)

Finally, to evaluate the more longitudinal trajectory estimation of the proposed method, the IMU measured data of one minutes, two minutes, and three minutes walks were analyzed, and the normalized endpoint error of estimated trajectories were evaluated. The route was same as the closed rectangular as shown in Figure 1(A) (e) and (B) (e), and the walk was continued until the predetermined walking time has elapsed. The trial number in each walk time were twice. Table 6 shows the results of evaluating endpoint error in the proposed method and the previous method.

**Table 6:** Results of the estimation error in the normalized endpoint position between the proposed method and the previous method with 1 min, 2 min, and 3 min walk.

Walk duration	Trial number	Data number	Normalized endpoint position [m]	
			Proposed method	Previous method
1 min	1st	46	0.00077	0.01493
	2nd	44	0.00461	0.01615
2 min	1st	88	0.00640	0.01152
	2nd	87	0.00611	0.01391
3 min	1st	128	0.00596	0.00968
	2nd	126	0.00267	0.00547

## Discussion

This study proposed a new method for estimating continuous gait trajectory by processing raw data of acceleration and angular velocity measured by a single shank-worn IMU. The accuracy of the proposed method is evaluated by correlation analysis based on simultaneous measurements with the golden standard. In segmental evaluation, the proposed method shows a high correlation with the golden standard in the stride length. The authors’ previous outcome^11^ which showed the mean accuracy (± standard deviation) was 0.054 ± 0.031 [m] for stride length was applied to estimate the local stepwise gait trajectory in the proposed method; similar experimental results were observed, showing that accuracy in median (1st quartile–3rd quartile) was − 0.027 (− 0.054 to − 0.006) [m] in this study. It is suggested that the proposed method has the same high accuracy as the previous outcomes. Further, the estimation method for the local stepwise gait trajectory can be extended continuously in this study. In the segmental evaluation, the proposed method showed a high correlation with the golden standard caused by the correlation analysis of the turning angle. The estimation error of turning angle in this study in median accuracy (1st quartile–3rd quartile) was 0.7 (− 0.2 to 1.7) [°], which suggests a relatively higher accuracy compared to the previous study^13^ reporting that estimation error of turning angle in mean accuracy (± standard deviation) was 1.6 ± 6.1 [°]. An evaluation experiment in the segmental evaluation is conducted under two walking speed conditions—normal speed and slow speed—considering the relatively slow walking speed of patients with gait disorders and healthy people. The correlation analysis results for stride length and turning angle between the proposed method and the golden standard for both the normal speed and slow speed conditions showed high correlation. Thus, it was considered robust against changes in walking speed. An evaluation experiment in the segmental evaluation was conducted with nine walking route conditions to comprehensively evaluate various applications including clinical walk test applications. The results showed a high correlation under all conditions; it is considered robust against route changes. Agreement between the two measurement methods, which were the proposed method and the gold standard, was evaluated by the Bland–Altman analysis against the stride length and turning angle in all experimental trials, along with the correlation analysis. In the Bland–Altman analysis, the result of evaluating LOA showed that more than 95% of the evaluated values in the analysis were included in the LOA. 95% of the error is contained around the LOA, which implies the two measurement methods are in agreement. Thus, the proposed method and the gold standard are considered equivalent.

Not only the segmental evaluation but also the entire evaluation was conducted because it was important for the continuous gait trajectory to evaluate the accuracy of the endpoint position error normalized by the number of strides between the proposed method and the gold standard. The result for the entire evaluation (0.029 (0.019–0.04) [m]) was comparable to the absolute value of results in the segmental evaluation regarding stride length (− 0.027 (− 0.054 to − 0.006) [m]). Further, the results were comparable to or less than those of the authors' previous report on local stepwise gait trajectory^11^ 0.054 ± 0.031 [m]. Thus, the accuracy of the proposed method in each gait cycle was maintained, and the endpoint position error of the proposed method was considered comparable to the stepwise evaluated accuracy. The results suggest that the proposed method is robust against estimating continuous gait trajectories in relatively long-distance measurements. In the walking route conditions in the entire evaluation, no specific trend was observed as a whole. Therefore, this no specific trend meant that the accuracy did not depend on the characteristics of a particular route, suggesting to be basically robust to various walking route conditions. However, the estimation error of normalized endpoint position in TUG3 route was slightly smaller compared to that in straight route as shown in Table 4. This is considered to be due to stride length estimation tendency. In the results of the Bland–Altman analysis shown in Figure 4, the stride length tends to be estimated slightly short of the golden standard. This error may be offset for TUG3M due to its circumferential route, but may not be offset for straight due to its one-way route. Therefore, the stride length estimation error is considered to have an effect. This is considered to be one of the issues to be addressed in the future. Under the walking speed conditions in the entire evaluation, the endpoint position error tended to be larger in the slow walking condition than in the normal walking condition. This comparison suggested that the accuracy in the slow walking condition was relatively lower than that in the normal condition. Even when considering the maximum value of the error in the slow walking condition, it was equal to or less than that in the previous report of the authors^11^. Therefore, it is considered sufficiently accurate based on this comparison.

Due to comparison of the proposed method to the representative previous method, the measured IMU dataset was applied to one of the conventional methods^5,24^. In the comparison results, the estimation errors of stride length in the proposed method (− 0.027 (− 0.054 to − 0.006) [m]) was comparable to the previous method (− 0.027 (− 0.053 to − 0.004) [m]). On the other hand, the variability which is range of estimation error of turning angle of the proposed method (0.7 (− 0.2–1.7) [°]) was much smaller than the previous method (0.3 (− 10.5–11.2) [°]). The estimation error of normalized endpoint position of the proposed method (0.029 (0.019–0.040) [m]) also improved compared to that of the previous method (0.052 (0.034–0.083) [m]). In the difference of the proposed method against the previous method implemented by using simple integral operation of angular velocity^5,24^, the calculation of turning angle was based on the integration of the stride vector angle and the shank orientation, meaning the usage not only angular velocity information but also local piecewise gait trajectory from acceleration information and the applications of geometric relationships between them. The stride vector angle was the angle of foot direction from certain stride to the next which was derived from the local piecewise gait trajectory on the local coordinate whose origin was set to that certain stride, based on an integration process of acceleration. The shank orientation from angular velocity defined the angler relationship between adjacent local coordinates of the local piecewise gait trajectory. Integration of both the angular change of a foot in a local coordinate and the derivation of the angular relationship between local coordinates was considered to contribute to higher accuracy of the turning angle, which in turn lead to an improvement in estimating endpoint position. Actually, in the longitudinal walking trial, the estimation accuracies in proposed method were nicer than that in previous method. The proposed method maintains the local stepwise gait trajectory in each gait cycle during walking and has been extended to the continuous gait trajectory as the 3D position time series in continuous walking; this suggests that the proposed method integrates the advantages of the global continuous gait path during walking and local stepwise gait trajectory during walking.

In comparison of some previous studies implementing gait trajectory and gait feature values estimation for clinical application^9,22^, 3D gait trajectory in the proposed method has potential to applicability for diagnosis with whole real gait movement related to any diseases^10,11^. Moreover, IMU mounting position may also have contributed to the low error. The area around the ankle has less soft tissue than the area around the foot, and the bone structure is relatively simple^18^. In additional comparison with other previous studies of the global continuous gait path measurement with shoes mounted IMU^3,4^, this proposed method does not use specific shoe between the IMU and body attachment points. This proposed method attached IMU on the shank to avoid the intervention of soft tissues, complex bone structures, and other shoes, being considered to maintain a low level of estimation error. Some methods exist that attempt to improve accuracy by using multiple sensors of different types, but many sensors need to be attached to shoes or other parts of the body^6^. The weight of the foot is considered to be one of the factors delaying social implementation, and the system should be configured to have as few parts as possible and be easy to use. The use of fewer and lighter sensors, as in the proposed method, does not slow down the deployability of future use cases.

When attempting to understand the walking motion, we must first focus on the shape and flow of the trajectory associated with the foot motion by visual inspection. The proposed method can help understand the positional relationship of the foot and trajectory as its history in an integrated manner because there is a one-to-one correspondence between the visible foot motion by visual inspection and the visualization of the continuous gait trajectory as an estimation result. It is a simple, low-cost, and highly accurate method for estimating the trajectory of walking motion by simply attaching a small ankle-sized sensor to the foot without using multiple external imaging devices that are fixed to the periphery of the measurement target as in the Golden Standard OMC. For clinical gait analysis, specific spatiotemporal parameters were important (e.g., initial/terminal contact time, foot clearance, stride length, gait speed, stride duration, stance duration, swing duration). The previous work^11^ has already proposed a method to estimate these parameters, which are mainly based on local piecewise gait trajectory estimation. However, the clinical gait assessment such as Performance Oriented Mobility Assessment (POMA) requires gait path straightness and includes the aspect of global continuous gait path. This kind of analyses of both local and global gait aspects must be linked to the possibility of further datafication and reuse of detailed diagnosis results. The proposed method was evaluated not only for healthy normal walking but also for the slow walking speed and clinical walk test specific walking route, which were considered applicable for clinical diagnosis. In the medical field, various diagnostic criteria have been proposed and are in use because of the accumulation of historical wisdom; the proposed method can be developed further and applied to elemental technologies that underpin its progress in evidence-based medicine and therapy.

There are some limitations of this study: The evaluation experiment of the proposed method was set up considering a clinical situation; however, the actual experimental participants were healthy young people. In the future, the reliability of the proposed method will need to be examined by applying this method to healthy elderly people and patients with actual inherent gait disorders. This study evaluated the trajectory estimation not only normal speed but also slow speed in assuming various types of walking. However, gait disorders show variety of symptoms such as circumduction gait and short-stepped gait. Further validation of the application to these various pathological gait patterns will be required. Further, the gender ratio of the experimental participants was not equal, and gender differences were not examined. These factors, as well as the age of the participants, will require additional investigation in future reliability studies. From the comparison of the experimental results in estimation error of normalized endpoint position of the straight route and the TUG3M route, it was possible that the trajectory estimation in a one-way path rather than a circumferential path might be affected by the estimation error of stride length. This is considered to be one of the issues to be addressed in the future. In the current study, the proposed method for estimating continuous gait trajectory is constructed by referring to the authors’ previous outcome, in which a parabolic regression model was used to identify and apply the segmentation feature points in the middle of the stance phase of the shank during walking. However, another method for estimating local stepwise gait trajectory during walking using an inverted pendulum model has been reported to further improve the accuracy and the robustness^10^. The method proposed in this study can be further developed to achieve higher accuracy and robustness in the future by applying such a method.

## Conclusion

We proposed a new estimation method of continuous gait trajectory during walking by processing raw data of acceleration and angular velocity measured from a single shank-worn IMU. We conducted evaluation experiments using the OMC as the golden standard considering its application in the medical field. The results of the evaluation experiment suggested high accuracy and good robustness in estimating stride length and turning angle in the segmental evaluation aspect, as well as in estimating the endpoint position of walking in the entire evaluation aspect. Further, the proposed method is expected to be applied in clinical practice and to contribute to daily life as an elemental technology for supporting the use of simple and low-cost high-precision gait motion information.

Beyond the medical field, it is expected to be applied to elucidate the characteristics of continuous or mid- to long-distance walking movements associated with unique sports and lifestyle that have not been analyzed so far because of the limitations of the measurement environment. Conventional lifelogging has attempted to accumulate the characteristics of gait movement; however, most of them have not dealt with continuous 3D gait trajectories. In the future, such trajectories of human gait are expected to be valuable, and this will expand the possibility of its application in social implementation.

## Proposed methods

### System setup

**Figure 8:**
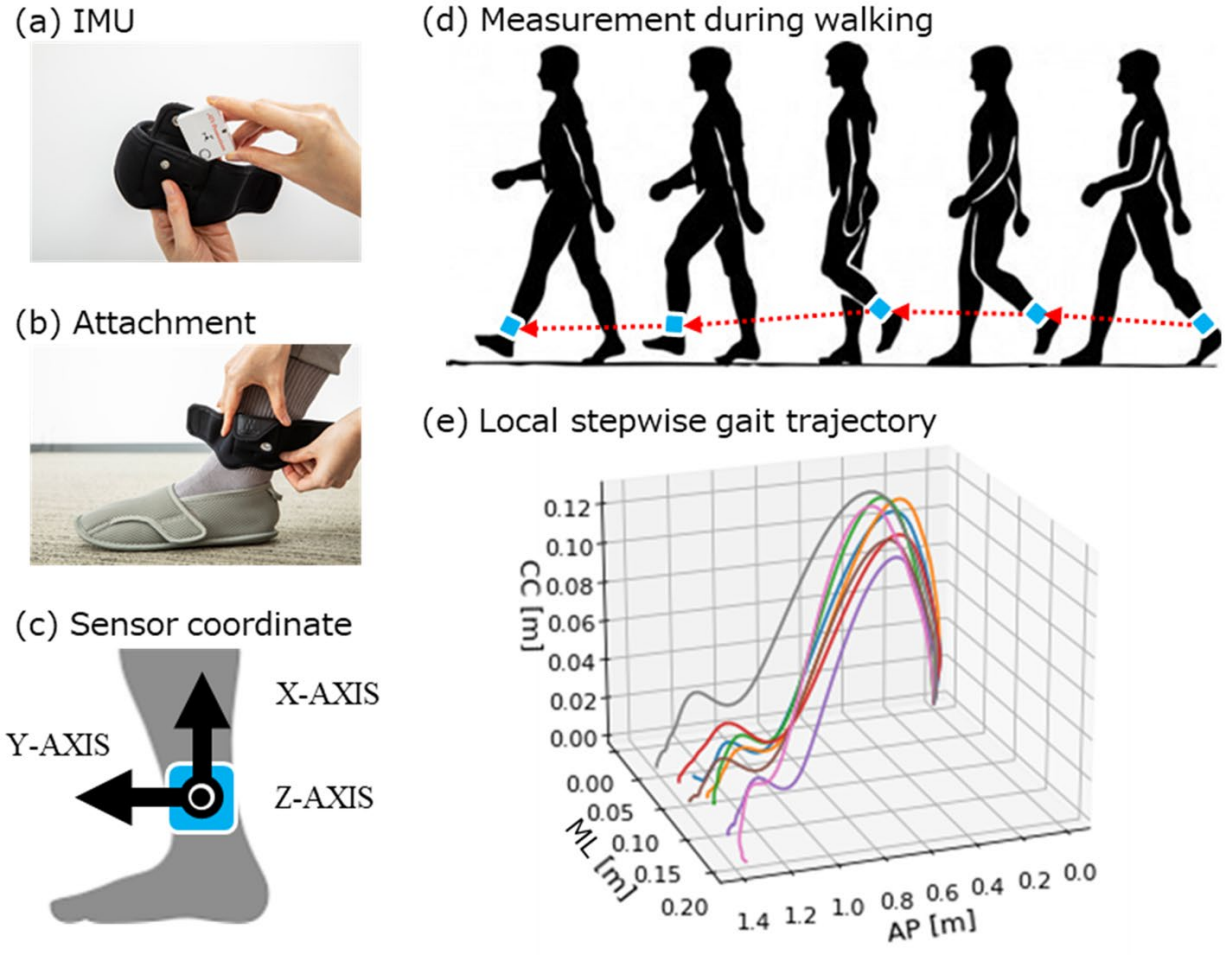
Outline of system setup in proposed method and example of local stepwise gait trajectory. (**a**) IMU sensor and an attachment equipment. (**b**) Attachment of the IMU sensor to the outside of the shank. (**c**) Setup of sensor coordinate system of the IMU sensor. The X, Y, and Z axes was set to craniocaudal direction, anterior–posterior direction, and medial–lateral direction. (**d**) Measurement of IMU data on the shank during walking. (**e**) Estimation example of local stepwise gait trajectory with IMU data on the shank during walking. In the axes, AP means anterior–posterior direction, ML means medial–lateral direction, and CC means craniocaudal direction.

**Figure 9:**
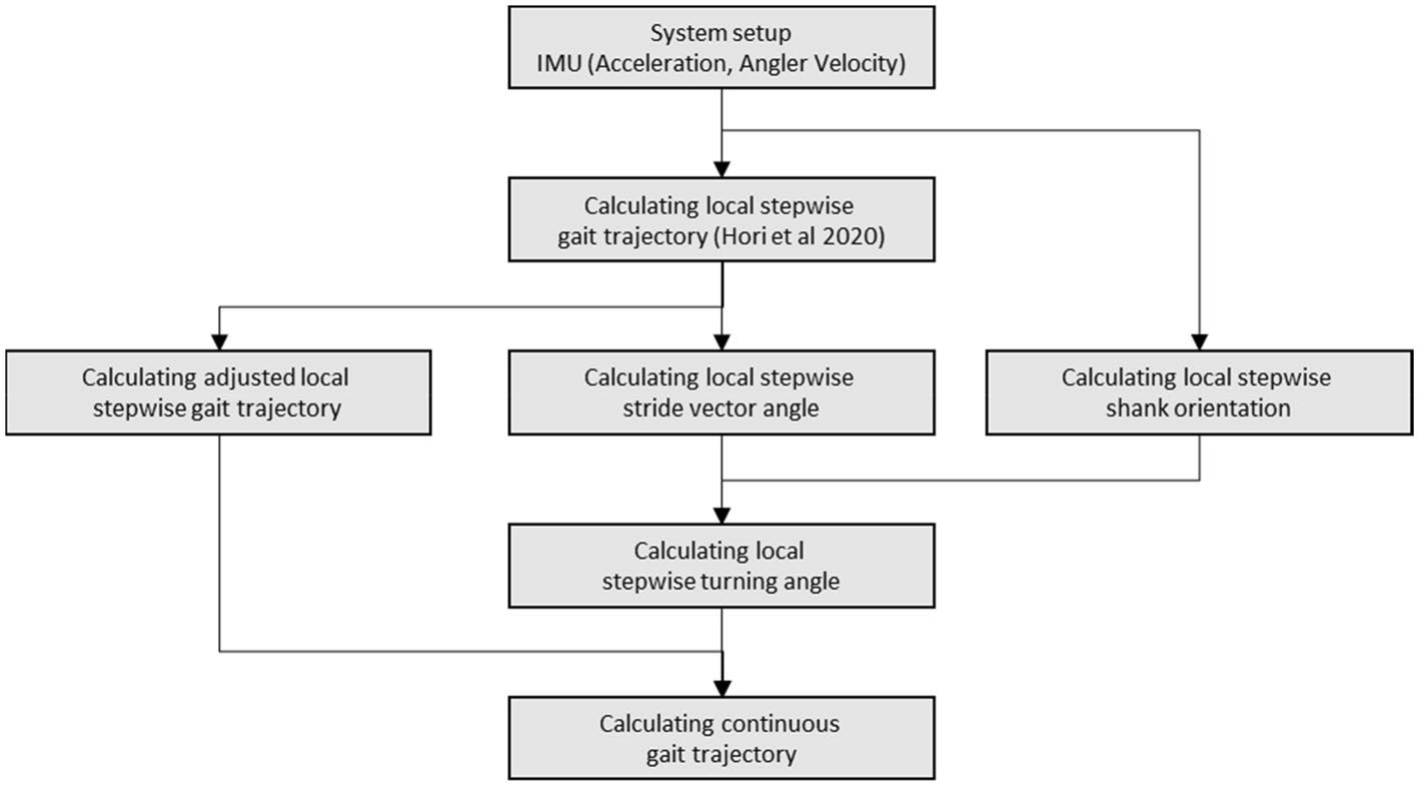
Flow chart of proposed method for estimating continuous gait trajectory using single shank-worn IMU.

An overview of the proposed method for estimating the continuous gait trajectory is presented in Fig. 8. The raw measurement data were taken by a single IMU attached to the shank in humans as shown in Fig. 8(a) and (b). The raw data measured by the IMU were the three-axis acceleration and three-axis angular velocity as shown in Fig. 8(c). In the sensor coordinate, the X, Y, and Z axes corresponded to the cranio-caudal, anterior–posterior, and medial–lateral directions, respectively. For details of the experimental system and experimental conditions, see < Experimental setup > subsection. The processing flow of the measured single IMU raw data is shown in Fig. 9. First, the local stepwise gait trajectory is estimated in 3D, and then, the adjacent trajectories are connected to estimate the continuous gait trajectory in 3D. To estimate the trajectories, this study followed a method previously proposed by the authors^11^.

### Calculate the local stepwise gait trajectory

A highly accurate local stepwise gait trajectory can be estimated for the shank during walking by acquiring the raw data of acceleration and angular velocity in the sensor coordinate from the IMU and applying the method outlined below, based on the previous study^11^. The characteristic time-series data waveform observed during the stance phase of walking was modeled using a parabola regression model; the data for each gait cycle were divided and cut out to estimate the stepwise shank trajectory, which suppress the accumulation of errors. The integral process in acceleration and velocity timeseries for each gait cycle is implemented by combining the integral calculation in the direction of the temporal forward with the integral calculation in the direction of temporal backward.

The raw data of acceleration ${\varvec{a}}^{S} \left( k \right)$ and angular velocity ${\varvec{\omega}}^{S} \left( k \right)$ in frame S as the sensor coordinate system are obtained from the IMU.

1 $$ \begin{array}{*{20}c} {a^{S} \left( k \right) = \left( {\begin{array}{*{20}c} {a_{x}^{S} \left( k \right)} \\ {a_{x}^{S} \left( k \right)} \\ {a_{x}^{S} \left( k \right)} \\ \end{array} } \right),\left( {k \in \left[ {0, N_{k} } \right),N \in {\mathbb{N}}} \right)} \\ \end{array} $$

2 $$ \begin{array}{*{20}c} {\omega^{S} \left( k \right) = \left( {\begin{array}{*{20}c} {\omega_{x}^{S} \left( k \right)} \\ {\omega_{x}^{S} \left( k \right)} \\ {\omega_{x}^{S} \left( k \right)} \\ \end{array} } \right), \left( {k \in \left[ {0, N_{k} } \right), N_{k} \in {\mathbb{N}}} \right)} \\ \end{array} $$

In Eqs. (1) and (2), (*k*) represents the *k-*th sample of an instantaneous variable, $N_{k}$ represents the total number of samples of an instantaneous variable, and ${\mathbb{N}}$ represents the set of natural numbers.

**Figure 10:**
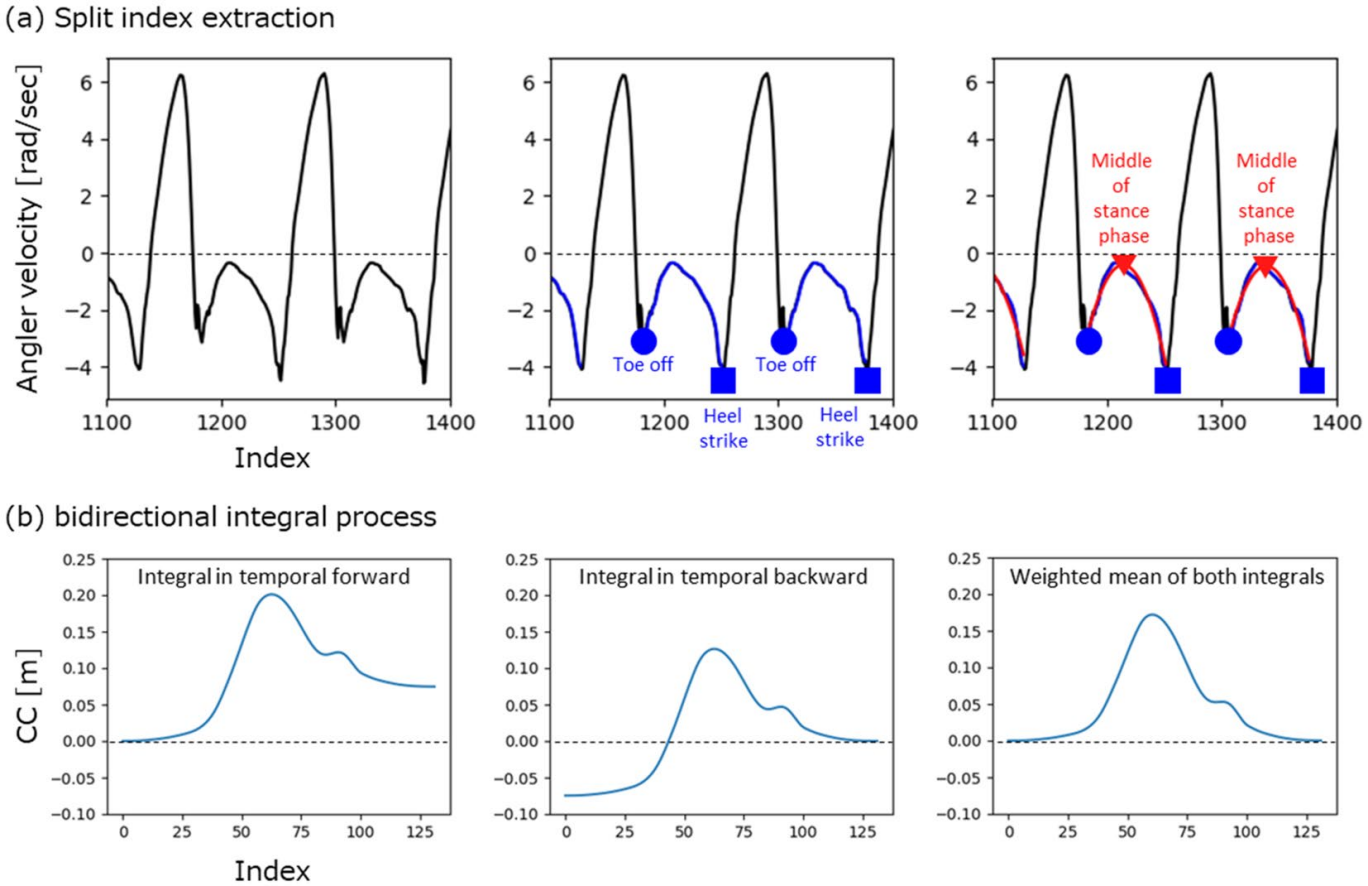
Process components for calculating local stepwise gait trajectory. (**a**) Example of split index extraction. Left part figure shows angular velocity of Z axis with median filter (window length: 5). Center part figure shows the timings of the toe off and the heel strike were extracted by searching the local maximum. Right part figure shows the parabolic regression model was applied to the angular velocity of stance phase which is between the toe off and the heel strike. (**b**) Example of bidirectional integral process with cranio-caudal component of trajectory. Left part figure is output trajectory from the integral calculation of velocity in the direction of temporal forward. Center part figure is output trajectory from the integral calculation of velocity in the direction of temporal backward. Right part figure is output of weighted mean of both outputs in the direction of temporal forward and temporal backward.

The events in the middle of the stance phase of the *i*-th gait cycle were estimated using the parabolic regression model as shown in Fig. 10(a). In angular velocity of Z axis with median filter (window length: 5) as shown in Fig. 10(a) left part, the timings of the toe off and the heel strike were extracted by searching the local maximum. After extracting the angular velocity between the toe off and the heel strike as stance phase of gait, the parabolic regression model was applied to the angular velocity of stance phase, as shown in Fig. 10(a) center part. The top point of the fitted parabolic regression model was adopted to the middle of the stance phase as the split point in the heading, as shown in Fig. 10(a) right part.

3 $$ \begin{array}{*{20}c} {ms\left( i \right), \left( {ms\left( i \right) \subset k,i \in \left[ {0,{ }N_{i} } \right),{ }N_{i} \in {\mathbb{N}},N_{i} < N_{k} } \right)} \\ \end{array} $$

where $ms\left( i \right)$ represents the index corresponding to the middle of the stance phase in index (*k*). ($N_{i}$) represents the total number of events in the middle of the stance phase.

The positional time series data ${\varvec{p}}^{E} \left( k \right)$ of the local stepwise gait trajectory at frame E was estimated using the raw data of acceleration ${\varvec{a}}^{S} \left( k \right)$ and angular velocity ${\varvec{\omega}}^{S} \left( k \right)$ and the index $ms\left( i \right)$. Where the frame E represents the world coordinate. In concrete, the raw data of acceleration ${\varvec{a}}^{S} \left( k \right)$ converted to the frame E’s acceleration, using the initial IMU posture based on direction of gravitational acceleration at the initial static part of acceleration raw data and IMU posture’s rotation change based on integral of the angular velocity with the initial IMU posture.

In each segmented based on the middle of the stance phase, the positional time series data ${\varvec{p}}^{E} \left( k \right)$ of the local stepwise gait trajectory at frame E was calculated by applying the bidirectional integral process to this frame E’s acceleration. To calculate the velocity, the bidirectional integral process was applied to each of the acceleration components in the three axis directions. To calculate the trajectory, it was applied to the velocity component in the cranio-caudal direction as shown in Fig. 10(b), and the simple integral process was applied to the velocity components in other directions.

The bidirectional integral process, for example of cranio-caudal component of trajectory, calculates the integral value as shown in Fig. 10(b) left part, also calculates the integral value from the end to the beginning of the input data as shown in Fig. 10 (b) center part, and then calculates the weighted mean of both integral values as shown in Fig. 10(b) right part. The example of the positional time series data ${\varvec{p}}^{E} \left( k \right)$ of the local stepwise gait trajectory at frame E, with walk as shown in Fig. 8(d), was the trajectories in Fig. 8(e). See previous study^11^ for further details.

4 $$ \begin{array}{*{20}c} {{\varvec{p}}^{E} \left( k \right) = \left( {\begin{array}{*{20}c} {p_{x}^{S} \left( k \right)} \\ {p_{x}^{S} \left( k \right)} \\ {p_{x}^{S} \left( k \right)} \\ \end{array} } \right), \left( {k \in \left[ {0,{ }N} \right),{ }N \in {\mathbb{N}}} \right)} \\ \end{array} $$

where

5 $$ \begin{array}{*{20}c} {{\varvec{p}}^{E} \left( {ms\left( i \right)} \right) = \left( {\begin{array}{*{20}c} {p_{x}^{S} \left( {ms\left( i \right)} \right)} \\ {p_{x}^{S} \left( {ms\left( i \right)} \right)} \\ {p_{x}^{S} \left( {ms\left( i \right)} \right)} \\ \end{array} } \right) = \left( {\begin{array}{*{20}c} 0 \\ 0 \\ 0 \\ \end{array} } \right)} \\ \end{array} $$

### Calculating adjusted local stepwise gait trajectory

The local stepwise gait trajectory is adjusted by introducing a new coordinate frame M to convert the local stepwise gait trajectory into a continuous gait trajectory. In frame M, the y-axis direction was aligned with the forward direction of each stride vector in the trajectory, and the sagittal plane was constructed by the y- and x-axes of frame E. Frame M was used to match the stride vectors for the local stepwise gait trajectories as adjusted stepwise gait trajectories. These adjusted local stepwise gait trajectories were used in the process described below to connect adjacent trajectories. In frame M, the y-axis direction was aligned with the forward direction, and the sagittal plane was constructed using the y- and x-axes of world frame E.

The three standard basis vectors of frame M are represented by three column vectors. This matrix is under frame E. This definition is necessary to obtain the rotation matrix from frame E to frame M.

6 $$ \begin{array}{*{20}c} {{\varvec{M}}^{E} \left( i \right) = \left[ {\begin{array}{*{20}c} {{\varvec{m}}_{x}^{E} \left( i \right)} & {{\varvec{m}}_{y}^{E} \left( i \right)} & {{\varvec{m}}_{z}^{E} \left( i \right)} \\ \end{array} } \right]} \\ \end{array} $$

where ${\varvec{M}}^{E} \left( i \right)$ represents the rotation matrix, and the vectors ${\varvec{m}}_{x}^{E} \left( i \right)$, ${\varvec{m}}_{y}^{E} \left( i \right)$, and ${\varvec{m}}_{z}^{E} \left( i \right)$ represent the standard basis vectors for the *i*-th gait cycle.

The direction of ${\varvec{m}}_{y}^{E} \left( i \right)$ is defined as the forward direction and is computed using the initial and final values of ${\varvec{p}}^{E} \left( k \right)$ in each gait cycle.

7 $$ \begin{array}{*{20}c} {{\varvec{m}}_{y}^{E} \left( i \right) = \frac{{{\varvec{p}}^{E} \left( {ms\left( {i + 1} \right) - 1} \right) - {\varvec{p}}^{E} \left( {ms\left( i \right)} \right)}}{{{\varvec{p}}^{E} \left( {ms\left( {i + 1} \right) - 1} \right) - {\varvec{p}}^{E} \left( {ms\left( i \right)} \right)}}} \\ \end{array} $$

Here, ${\varvec{m}}_{z}^{E} \left( i \right)$ is defined as a vector perpendicular to the plane determined by ${\varvec{m}}_{y}^{E} \left( i \right)$ and the x-axis of frame E. It was computed and normalized using the outer product.

8 $$ \begin{array}{*{20}c} {{\varvec{m}}_{z}^{E} \left( i \right) = \frac{{{\varvec{e}}_{x} \times {\varvec{y}}^{E} \left( i \right)}}{{{\varvec{e}}_{x} \times {\varvec{y}}^{E} \left( i \right)}}} \\ \end{array} $$

The unit vectors for the x-, y-, and z-axes are

9 $$ \begin{array}{*{20}c} {{\varvec{e}}_{x} = \left( {\begin{array}{*{20}c} 1 & 0 & 0 \\ \end{array} } \right)^{T} , \user2{ e}_{y} = \left( {\begin{array}{*{20}c} 0 & 1 & 0 \\ \end{array} } \right)^{T} , \user2{ e}_{z} = \left( {\begin{array}{*{20}c} 0 & 0 & 1 \\ \end{array} } \right)^{T} } \\ \end{array} $$

Next, ${\varvec{m}}_{x}^{E} \left( i \right)$ was obtained from the outer product of ${\varvec{m}}_{z}^{E} \left( i \right)$ and ${\varvec{m}}_{y}^{E} \left( i \right)$.

10 $$ \begin{array}{*{20}c} {{\varvec{m}}_{x}^{E} \left( i \right) = {\varvec{m}}_{y}^{E} \left( i \right) \times {\varvec{m}}_{z}^{E} \left( i \right)} \\ \end{array} $$

where the rotation matrix of frame M, represented below frame M, is a 3 × 3 unit matrix $I_{3}$, where the main diagonal elements are equal to 1.

11 $$ \begin{array}{*{20}c} {I_{3} = \left( {\begin{array}{*{20}c} 1 & 0 & 0 \\ 0 & 1 & 0 \\ 0 & 0 & 1 \\ \end{array} } \right)} \\ \end{array} $$

Therefore, the rotation matrix ${\varvec{R}}^{EM} \left( i \right)$ was derived by solving

12 $$ \begin{array}{*{20}c} {{\varvec{I}}_{3} = {\varvec{R}}^{EM} \left( i \right) \cdot M^{E} \left( i \right)} \\ \end{array} $$

The adjusted local stepwise gait trajectory ${\varvec{p}}^{M} \left( k \right)$ for frame M was transformed using the rotation matrix applied to ${\varvec{p}}^{E} \left( k \right)$ in a segmented manner.

13 $$ \begin{array}{*{20}c} {{\varvec{p}}^{M} \left( k \right) = {\varvec{R}}^{EM} \left( i \right) \cdot {\varvec{p}}^{E} \left( k \right), k \in \left[ {ms\left( i \right), ms\left( {i + 1} \right)} \right)} \\ \end{array} $$

### Calculating local stepwise shank orientation

**Figure 11:**
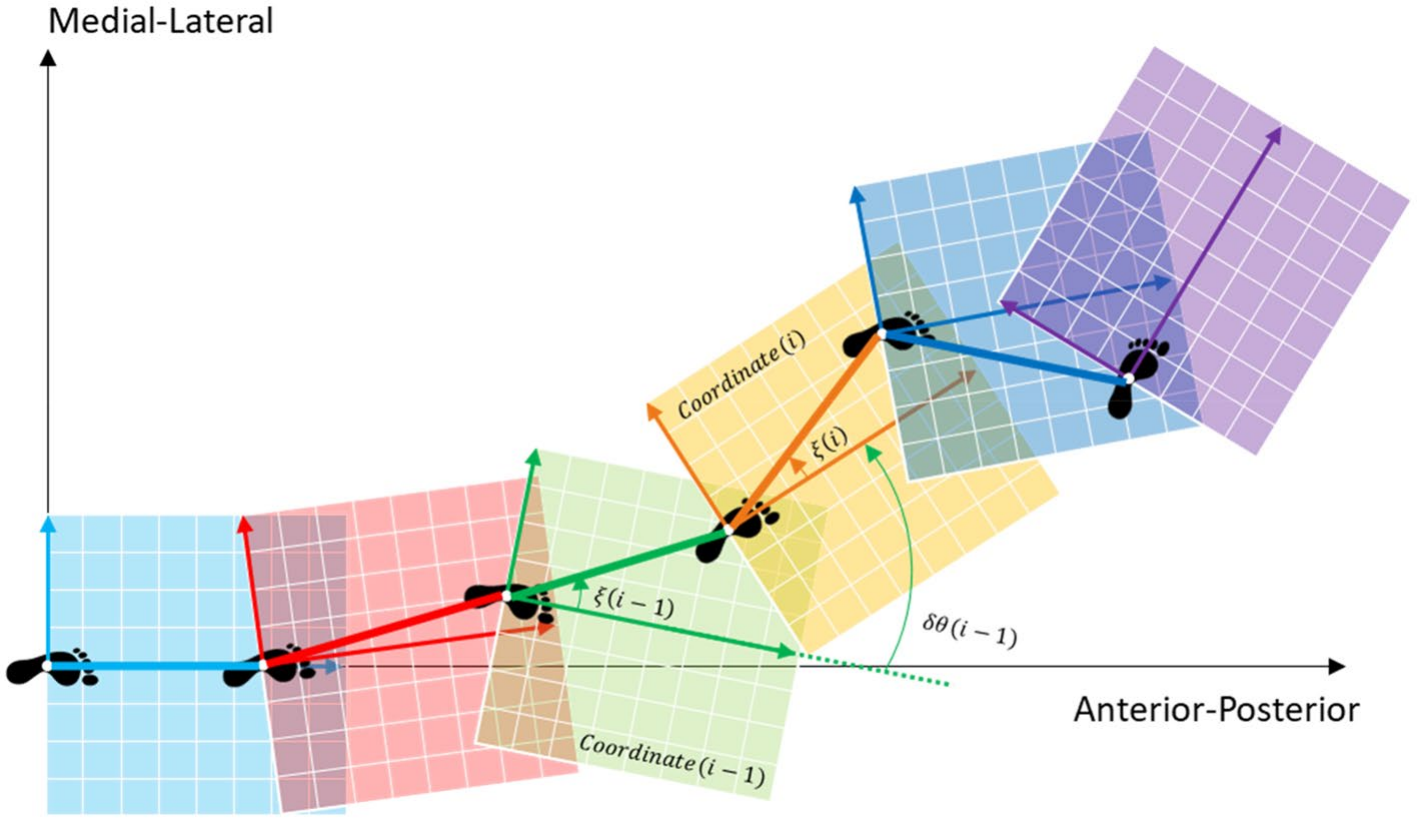
Relationship between adjacent local stepwise gait trajectories.

It is necessary to derive the local stepwise shank orientation $\xi \left( i \right)$ and local stepwise stride vector angle $\delta \theta \left( i \right)$ in the horizontal plane to transform the adjusted local stepwise gait trajectory ${\varvec{p}}^{M} \left( k \right)$ into a continuous gait trajectory, as shown in Fig. 11. To derive these, the posture of the shank is estimated using the Euler angle from the IMU data.

The rotation matrix ${\varvec{R}}^{{{\varvec{SE}}}} \left( i \right)$ for each gait cycle transforming from frame S to frame E is obtained as

14 $$ \begin{aligned} {\varvec{R}}^{{{\varvec{SE}}}} \left( i \right) = & {\varvec{R}}_{{\varvec{x}}} \left( {\theta \left( i \right)} \right)\user2{ R}_{{\varvec{y}}} \left( {\varphi \left( i \right)} \right) {\varvec{R}}_{{\varvec{z}}} \left( {\psi \left( i \right)} \right) \\ = & \left( {\begin{array}{*{20}l} {\cos \varphi \left( i \right)\cos \psi \left( i \right)} \hfill & { - \cos \varphi \left( i \right)\sin \psi \left( i \right)} \hfill & {\sin \varphi \left( i \right)} \hfill \\ {\sin \theta \left( i \right)\sin \varphi \left( i \right)\cos \psi \left( i \right) + \cos \theta \left( i \right)\sin \psi \left( i \right)} \hfill & { - \sin \theta \left( i \right)\sin \varphi \left( i \right)\sin \psi \left( i \right) + \cos \theta \left( i \right)\cos \psi \left( i \right)} \hfill & { - \sin \theta \left( i \right)\cos \varphi \left( i \right)} \hfill \\ { - \cos \theta \left( i \right)\sin \varphi \left( i \right)\cos \psi \left( i \right) + \sin \theta \left( i \right)\sin \varphi \left( i \right)} \hfill & {\cos \theta \left( i \right)\sin \varphi \left( i \right)\sin \psi \left( i \right) + \sin \theta \left( i \right)\cos \psi \left( i \right)} \hfill & {\cos \theta \left( i \right)\cos \varphi \left( i \right)} \hfill \\ \end{array} } \right) \\ \end{aligned} $$

where the Euler angle is given by the Z-Y-X rotation. Here, ${\varvec{R}}_{{\varvec{x}}} \left( \theta \right)$, ${\varvec{R}}_{{\varvec{y}}} \left( \varphi \right)$, and ${\varvec{R}}_{{\varvec{z}}} \left( \psi \right)$ are defined as

15 $$ \begin{array}{*{20}c} {{\varvec{R}}_{{\varvec{x}}} \left( \theta \right) = \left( {\begin{array}{*{20}c} 1 & 0 & 0 \\ 0 & {\cos \theta } & { - \sin \theta } \\ 0 & {\sin \theta } & {\cos \theta } \\ \end{array} } \right)} \\ \end{array} $$

16 $$ \begin{array}{*{20}c} {{\varvec{R}}_{{\varvec{y}}} \left( \varphi \right) = \left( {\begin{array}{*{20}c} {\cos \varphi } & 0 & {\sin \varphi } \\ 0 & 1 & 0 \\ { - \sin \varphi } & 0 & {\cos \varphi } \\ \end{array} } \right)} \\ \end{array} $$

17 $$ \begin{array}{*{20}c} {{\varvec{R}}_{{\varvec{z}}} \left( \psi \right) = \left( {\begin{array}{*{20}c} {\cos \varphi } & { - \sin \varphi } & 0 \\ {\sin \varphi } & {\cos \varphi } & 0 \\ 0 & 0 & 1 \\ \end{array} } \right)} \\ \end{array} $$

The initial Euler angles were calculated for each gait cycle. At the beginning of each cycle, i.e., at $k = ms\left( i \right)$, the foot is in full contact with the floor and can be assumed to be temporarily stationary. The accelerometer is assumed to initially detect only gravitational acceleration g.

18 $$ \begin{array}{*{20}c} {{\varvec{R}}^{SE} \left( i \right){\varvec{a}}^{S} \left( {ms\left( i \right)} \right) = g} \\ \end{array} $$

19 $$ \begin{array}{*{20}c} { \Leftrightarrow {\varvec{a}}^{S} \left( {ms\left( i \right)} \right) = {\varvec{R}}^{SE} \left( i \right)^{ - 1} g} \\ \end{array} $$

20 $$ \begin{array}{*{20}c} { \Leftrightarrow {\varvec{a}}^{S} \left( {ms\left( i \right)} \right) = {\varvec{R}}^{SE} \left( i \right)^{T} g} \\ \end{array} $$

Here,

21 $$ \begin{array}{*{20}c} {{\varvec{R}}^{SE} \left( i \right)^{ - 1} = {\varvec{R}}^{SE} \left( i \right)^{T} } \\ \end{array} $$

22 $$ \begin{array}{*{20}c} {g = \left( {\begin{array}{*{20}c} { - 1} \\ 0 \\ 0 \\ \end{array} } \right)} \\ \end{array} $$

23 $$ \begin{aligned} {\varvec{a}}^{S} \left( {ms\left( i \right)} \right) = & \left( {\begin{array}{*{20}c} {{\varvec{a}}_{{\varvec{x}}}^{{\varvec{S}}} \left( {ms\left( i \right)} \right)} \\ {{\varvec{a}}_{{\varvec{y}}}^{{\varvec{S}}} \left( {ms\left( i \right)} \right)} \\ {{\varvec{a}}_{{\varvec{z}}}^{{\varvec{S}}} \left( {ms\left( i \right)} \right)} \\ \end{array} } \right) = {\varvec{R}}^{SE} \left( i \right)^{T} {\mathbf{g}} \\ = & \left( {\begin{array}{*{20}l} {\cos \varphi \left( i \right)\cos \psi \left( i \right)} \hfill & {\sin \theta \left( i \right)\sin \varphi \left( i \right)\cos \psi \left( i \right) + \cos \theta \left( i \right)\sin \psi \left( i \right)} \hfill & { - \cos \theta \left( i \right)\sin \varphi \left( i \right)\cos \psi \left( i \right) + \sin \theta \left( i \right)\sin \varphi \left( i \right)} \hfill \\ { - \cos \varphi \left( i \right)\sin \psi \left( i \right)} \hfill & { - \sin \theta \left( i \right)\sin \varphi \left( i \right)\sin \psi \left( i \right) + \cos \theta \left( i \right)\cos \psi \left( i \right)} \hfill & {\cos \theta \left( i \right)\sin \varphi \left( i \right)\sin \psi \left( i \right) + \sin \theta \left( i \right)\cos \psi \left( i \right)} \hfill \\ {\sin \varphi \left( i \right)} \hfill & { - \sin \theta \left( i \right)\cos \varphi \left( i \right)} \hfill & {\cos \theta \left( i \right)\cos \varphi \left( i \right)} \hfill \\ \end{array} } \right)\left( {\begin{array}{*{20}c} { - 1} \\ 0 \\ 0 \\ \end{array} } \right) \\ & = \left( {\begin{array}{*{20}c} { - \cos \varphi \left( i \right)\cos \psi \left( i \right)} \\ {\cos \varphi \left( i \right)\sin \psi \left( i \right)} \\ { - \sin \varphi \left( i \right)} \\ \end{array} } \right) \\ \end{aligned} $$

Therefore, at $ms\left( i \right)$, the initial Euler angle vector ${\varvec{\theta}}_{{{\varvec{ms}}}} \left( i \right)$ as the shank posture is calculated from the IMU data as

24 $$ \begin{array}{*{20}c} {{\varvec{\theta}}_{{{\varvec{ms}}}} \left( i \right) = \left( {\begin{array}{*{20}c} {\theta \left( i \right)} \\ {\varphi \left( i \right)} \\ {\psi \left( i \right)} \\ \end{array} } \right) = \left( {\begin{array}{*{20}l} 0 \hfill \\ {\tan^{ - 1} \left( {\frac{{ - a_{z}^{S} \left( {ms\left( i \right)} \right)}}{{\sqrt {a_{x}^{S} \left( {ms\left( i \right)} \right)^{2} + a_{y}^{S} \left( {ms\left( i \right)} \right)^{2} } }}} \right)} \hfill \\ {\tan^{ - 1} \left( {\frac{{ - a_{y}^{S} \left( {ms\left( i \right)} \right)}}{{a_{x}^{S} \left( {ms\left( i \right)} \right)}}} \right)} \hfill \\ \end{array} } \right)} \\ \end{array} $$

where $\theta \left( i \right)$, $\varphi \left( i \right)$, and $\psi \left( i \right)$ are the initial Euler angles around the x-, y-, and z-axes, respectively.

Next, the time derivative of the Euler angle is calculated to obtain the Euler angle over time.

The relationship between the angular velocity ${\varvec{\omega}}\left( k \right)$ and time derivative of the Euler angle $\dot{\user2{\theta }}\left( k \right)$ can be calculated as

25 $$ \begin{array}{*{20}c} {\dot{\user2{\theta }}\left( k \right) = \left( {\begin{array}{*{20}c} {\dot{\theta }\left( k \right)} \\ {\dot{\varphi }\left( k \right)} \\ {\dot{\psi }\left( k \right)} \\ \end{array} } \right) = \left( {\begin{array}{*{20}l} {\frac{\cos \psi \left( k \right)}{{\cos \varphi \left( k \right)}}} \hfill & {\frac{ - \sin \psi \left( k \right)}{{\cos \varphi \left( k \right)}}} \hfill & 0 \hfill \\ {\sin \psi \left( k \right)} \hfill & {\cos \psi \left( k \right)} \hfill & 0 \hfill \\ { - \tan \varphi \left( k \right)\cos \psi \left( k \right)} \hfill & {\tan \varphi \left( k \right)\sin \psi \left( k \right)} \hfill & 1 \hfill \\ \end{array} } \right)\omega \left( k \right)} \\ \end{array} $$

The Euler angles are calculated by integration as

26 $$ \begin{array}{*{20}c} {\theta \left( k \right) = \left( {\begin{array}{*{20}c} {\theta \left( k \right)} \\ {\varphi \left( k \right)} \\ {\psi \left( k \right)} \\ \end{array} } \right) = \left\{ {\begin{array}{l} {{\varvec{\theta}}_{{{\varvec{ms}}}} \left( i \right), k = ms\left( i \right)} \\ \\ {{\varvec{\theta}}_{{{\varvec{ms}}}} \left( i \right) + \mathop \sum \limits_{j = ms\left( i \right) + 1}^{k} \dot{\user2{\theta }}\left( j \right){\Delta }t, ms\left( i \right) < k < ms\left( {i + 1} \right)} \\ \end{array} } \right.} \\ \end{array} $$

where ${\Delta }t$ denotes the sampling interval. When ${\varvec{k}} = {\varvec{ms}}\left( i \right)$, ${\varvec{\theta}}\left( k \right) = {\varvec{\theta}}_{{{\varvec{ms}}}} \left( i \right)$ is applied preferentially.

### Calculation of the local stepwise stride vector angle

The horizontal angle between the stride vector and forward direction in each gait cycle ξ(i) is

27 $$ \begin{array}{*{20}c} {\xi \left( i \right) = \tan^{ - 1} \left( {\frac{{p_{y}^{E} \left( { ms\left( {i + 1} \right) - 1} \right) - p_{y}^{E} \left( { ms\left( i \right) } \right)}}{{p_{z}^{E} \left( { ms\left( {i + 1} \right) - 1} \right) - p_{z}^{E} \left( { ms\left( i \right) } \right)}}} \right)} \\ \end{array} $$

The horizontal angle of the shank orientation in each gait cycle $\delta \theta \left( i \right)$ is expressed as

28 $$ \begin{array}{*{20}c} {\delta \theta \left( i \right) = \theta \left( {ms\left( {i + 1} \right) - 1} \right) - \theta \left( {ms\left( i \right)} \right)} \\ \end{array} $$

**Figure 12:**
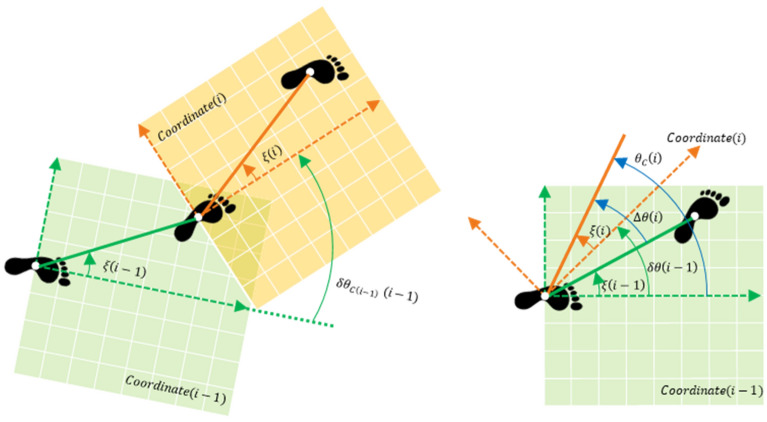
Relationship among local stepwise shank orientation $\xi \left( i \right)$, local stepwise stride vector angle $\delta \theta \left( i \right)$, and local stepwise turning angle $\theta_{C} \left( i \right)$ in horizontal plane for calculating continuous gait trajectory.

Based on these $\xi \left( i \right)$ and $\delta \theta \left( i \right)$, the angle between adjacent stride vectors in each gait cycle $\Delta \theta \left( {\text{i}} \right)$ is given as (Fig. 12)

29 $$ \begin{array}{*{20}c} {\Delta \theta \left( {\text{i}} \right) = \xi \left( i \right) + \left( {\delta \theta \left( {i - 1} \right) - \xi \left( {i - 1} \right)} \right),\;where \Delta \theta \left( 0 \right) = 0} \\ \end{array} $$

### Calculating local stepwise turning angle

Frame C was introduced as a coordinate for constructing a continuous gait trajectory. Frame C is a coordinate with the y-axis for the forward direction, z-axis for the medial–lateral direction, and x-axis for the up-down direction, of the shank at $i = 0$. The local stepwise turning angle in the horizontal plane that the stride vector in each gait cycle makes with the y-axis of frame C is given by $\theta_{C} \left( i \right)$ as

30 $$ \begin{array}{*{20}c} {\theta_{C} \left( i \right) = \theta_{C} \left( {i - 1} \right) + \Delta \theta \left( {\text{i}} \right),\;where\,\, \theta_{C} \left( 0 \right) = 0} \\ \end{array} $$

The rotation matrix ${\varvec{R}}^{{{\varvec{MC}}}} \left( i \right)$ for the coordinate transformation from frame M to frame P is

31 $$ \begin{aligned} {\varvec{R}}^{{{\varvec{MC}}}} \left( i \right) &=  {\varvec{R}}_{{\varvec{x}}} \left( \theta \right)\user2{ R}_{{\varvec{y}}} \left( \varphi \right) {\varvec{R}}_{{\varvec{z}}} \left( \psi \right) \\ &=  {\varvec{R}}_{{\varvec{x}}} \left( {\theta_{C} \left( i \right)} \right) {\varvec{R}}_{{\varvec{y}}} \left( 0 \right) {\varvec{R}}_{{\varvec{z}}} \left( 0 \right) = \left( {\begin{array}{*{20}c} 1 & 0 & 0 \\ 0 & {\cos \theta_{C} \left( i \right)} & { - \sin \theta_{C} \left( i \right)} \\ 0 & {\sin \theta_{C} \left( i \right)} & {\cos \theta_{C} \left( i \right)} \\ \end{array} } \right) \\ \end{aligned} $$

where $\varphi = 0$ and $\psi = 0$ because of handling only horizontal rotation.

### Calculate continuous gait trajectory

The translation vectors of the y- and z-axes of the stride vectors in each gait cycle were calculated for use as bias components. The stride length for each gait cycle $l\left( i \right)$ is calculated as

32 $$ \begin{array}{*{20}c} {l\left( i \right) = {\varvec{p}}^{E} \left( {ms\left( {i + 1} \right) - 1} \right) - {\varvec{p}}^{E} \left( {ms\left( i \right)} \right)} \\ \end{array} $$

The stride length in each gait cycle $l\left( i \right)$ is decomposed into the components of the y-axis in the forward direction and the z-axis in the medial–lateral direction based on $\theta_{C} \left( i \right)$.

33 $$ \begin{array}{*{20}c} {{{\varvec{\Delta}}}{\varvec{b}}^{{{\varvec{MC}}}} \left( i \right) = \left( {\begin{array}{*{20}c} 0 \\ {l\left( i \right)\cos \theta_{C} \left( i \right)} \\ {l\left( i \right)\sin \theta_{C} \left( i \right)} \\ \end{array} } \right)} \\ \end{array} $$

Therefore, the bias in the y-axis in the forward direction and the z-axis in the medial–lateral direction at frame C can be obtained as

34 $$ \begin{array}{*{20}c} {{\varvec{b}}^{{{\varvec{MC}}}} \left( i \right) = {\varvec{b}}^{{{\varvec{MC}}}} \left( {i - 1} \right) + {{\varvec{\Delta}}}{\varvec{b}}^{{{\varvec{MC}}}} \left( i \right)} \\ \end{array} $$

The rotation and parallel movements are represented by affine matrices. Based on the rotation matrix ${\varvec{R}}^{{{\varvec{MC}}}} \left( i \right)$ and parallel translation vector ${\varvec{b}}^{{{\varvec{MC}}}} \left( i \right)$, the affine matrix to be applied to the i-th walking trajectory in frame M is constructed as

35 $$ \begin{array}{*{20}c} {{\varvec{A}}^{{{\varvec{MC}}}} \left( i \right) = \left( {\begin{array}{*{20}c} {{\varvec{R}}^{{{\varvec{MC}}}} \left( i \right)} & {{\varvec{b}}^{{{\varvec{MC}}}} \left( {i - 1} \right)} \\ {{\varvec{o}}^{{\varvec{T}}} } & 1 \\ \end{array} } \right)} \\ \end{array} $$

where

36 $$ \begin{array}{*{20}c} {{\varvec{A}}^{{{\varvec{MC}}}} \left( 0 \right) = E = \left( {\begin{array}{*{20}c} 1 & 0 & 0 & 0 \\ 0 & 1 & 0 & 0 \\ 0 & 0 & 1 & 0 \\ 0 & 0 & 0 & 1 \\ \end{array} } \right)} \\ \end{array} $$

The position time series data ${\varvec{p}}^{C} \left( k \right)$ of the continuous walking trajectory is derived by applying ${\varvec{A}}^{{{\varvec{MC}}}} \left( {\text{i}} \right)$ to ${\varvec{p}}^{M} \left( k \right)$, where ${\varvec{p}}^{C} \left( k \right)$ and ${\varvec{p}}^{M} \left( k \right)$ are both converted into a four-element vector for the convenience of matrix calculation of the affine transformation using a 4 × 4 affine matrix.

37 $$ \begin{array}{*{20}c} {\left( {\begin{array}{*{20}c} {{\varvec{p}}^{C} \left( k \right)} \\ 1 \\ \end{array} } \right) = {\varvec{A}}^{{{\varvec{MC}}}} \left( {\text{i}} \right) \cdot \left( {\begin{array}{*{20}c} {{\varvec{p}}^{M} \left( k \right)} \\ 1 \\ \end{array} } \right), k \in \left[ {ms\left( i \right), ms\left( {i + 1} \right)} \right)} \\ \end{array} $$

## Evaluation experiment

### Experimental participants

A total of 12 participants participated in the evaluation experiment. Table 1 shows the characteristics of the participants. The study was conducted in accordance with the Declaration of Helsinki. This experiment was approved by the Ethics Committee of the Tokyo Institute of Technology. Written informed consent was obtained from all participants.

### Experimental conditions

**Figure 13:**
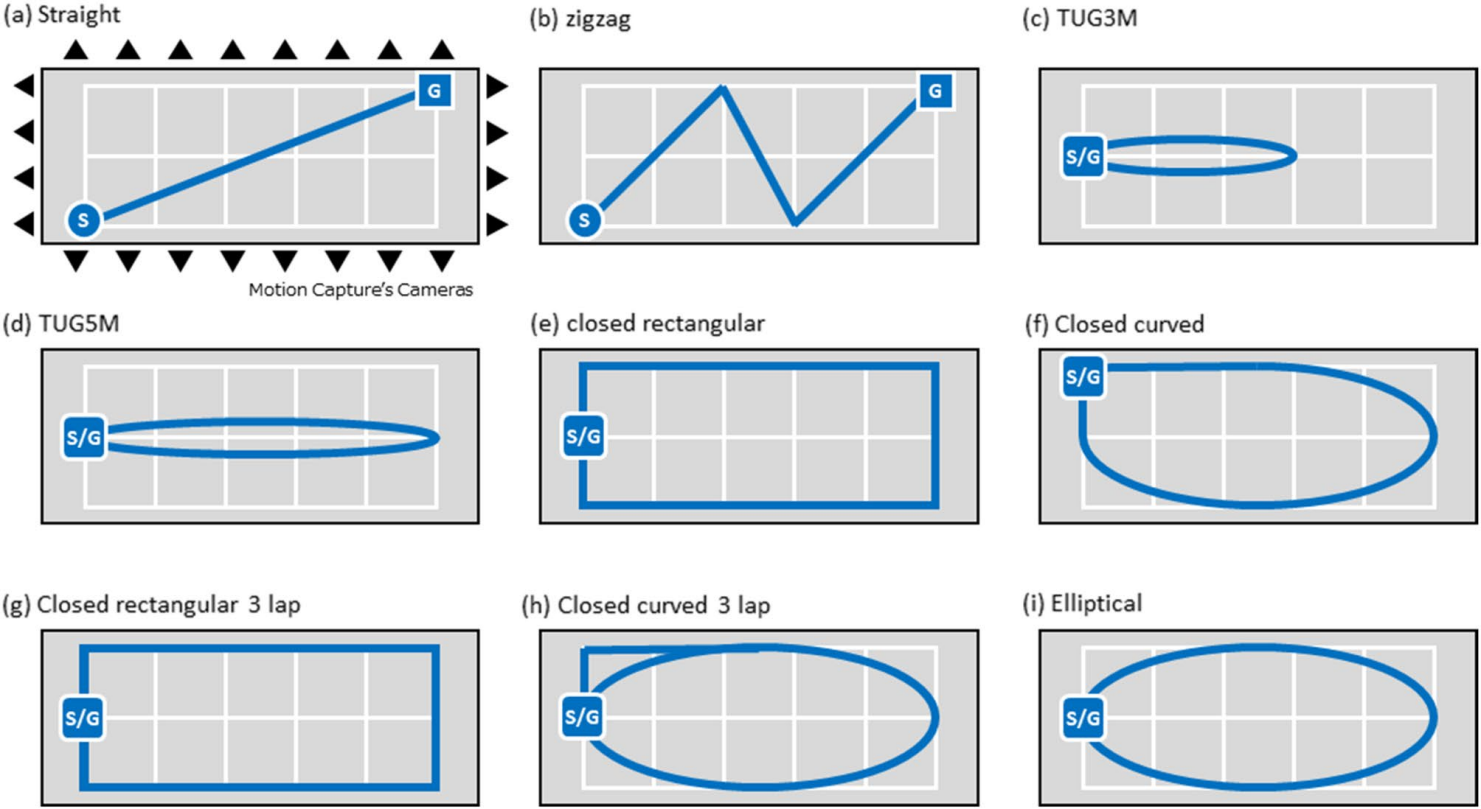
Nine walking route experimental conditions: (**a**) straight, (**b**) zigzag, (**c**) TUG3M, (**d**) TUG5M, (**e**) closed rectangular, (**f**) closed curved, (**g**) closed rectangular 3 lap, (**h**) closed curved 3 lap, and (**i**) elliptical.

Figure 13 shows that the walking experiments are performed under nine different walking route conditions. The nine types of walking route conditions include straight, zigzag, TUG3M, TUG5M, closed rectangular, closed curved, closed rectangular 3 lap, closed curved 3 lap, and elliptical.

The straight-route condition is the most basic walking route condition, and it is used clinically during clinical walk tests related to the 10 m walk test (10 MWT), 5 m walk test (5 MWT), and dynamic gait index (DGI). The zigzag route condition is a Z-shaped walking route that includes two sharp turns. The TUG3M (3 m timed up and go) route condition is a 3 m round trip walking route condition with a turning point set at 3 m in the forward direction from the starting point. TUG (timed up and go) is one of the clinical walk tests. The TUG5M (5 m timed up and go) route condition is a 5 m round-trip walking route condition with the same concept as TUG3M; however, for this route, the turning point is set at 5 m in the forward direction from the starting point.

Originally, the TUG was used as a clinical walk test to measure the time from sitting on a chair to standing up, returning to the chair after the turning point, and finally sitting down. It was used to evaluate the complex movements of daily activities such as standing, sitting, and walking in a straight line and turning. However, in this study, these TUG related conditions focus only on walking, and therefore, the test started and ended in an upright position, rather than in a sitting position.

The closed-rectangular route condition is a closed-circumferential route composed of a rectangle. The closed-curved route condition is a closed-circumferential route in which all corner parts of the rectangular path condition, except for the start and end points of the walk, are composed of smooth curved lines. The closed-rectangular 3 lap route condition includes three laps of the closed-rectangular route condition. The closed-curved 3 lap route condition includes three laps of the closed-curved route condition. The closed-elliptical route condition is a closed-circular route configured in an elliptical shape. The closed-elliptical route condition differs from the closed-curved route condition in that it is a closed-circumferential route in which all corner parts—including the start and end points of the walk in the closed-rectangular route condition—are composed of smooth curved lines.

The participants performed the walking tasks based on the above nine walking route conditions in a certain order at two different walking speeds of their choice: normal and slow. In the future, we envision that this system will be used for elderly people and people with gait disabilities in cerebral nervous system diseases such as Parkinson’s disease and/or a musculoskeletal disease such as arthropathy. Since walking speed decreases with age and disability^15,23^, we employed both the slow walking and normal speed conditions.

The walking speed conditions that include not only the normal speed condition but also the slow speed condition are set as experimental conditions different from the walking route conditions. All participants performed the experiment once under each condition.

### Experimental setup

The participants' walking was measured simultaneously with one IMU as the proposed method and the OMC as the golden standard to evaluate the accuracy of the proposed method. An overview is shown in Fig. 8(a)–(c).

Raw measurement data were obtained using one IMU (TSND151, ATR-Promotions, Kyoto, Japan) attached to the left shank of an experimental participant. The size of the IMU was approximately 40 mm (W) × 50 mm (H) × 14 mm (D), and the weight was approximately 27 g. The IMU was placed in a housing pocket equipped with a rubber band worn around the shank. The rubber band with the IMU in the storage pocket was attached at a position 0.03 cm above the ankle of the tibia.

Raw data measured by the IMU were acceleration in the three XYZ axes (± 8 G range) and angular velocity in the three XYZ axes (± 1,000°/s (DPS) range); the sampling frequency was 100 Hz for the IMU. In the sensor coordinate system, the X, Y, and Z axes correspond to the cranio-caudal, anterior–posterior, and medial–lateral directions, respectively. The IMU was connected to a laptop PC (Lenovo, ThinkPad T480s, Window10) via a Bluetooth protocol connection, and the raw data were stored on a laptop PC using ALTIMA (ATR-Promotions, Windows), which is a dedicated software for IMU data transmission and reception.

**Figure 14:**
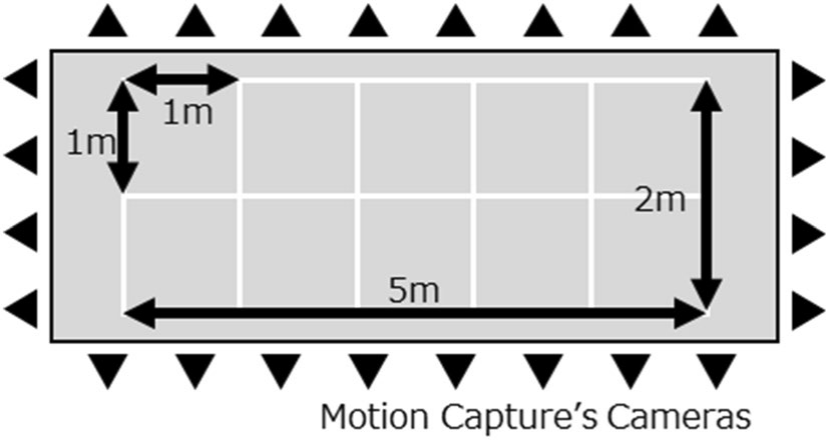
Experimental environment for participant’s walking with optical motion capture system including 24 cameras.

The walking movement of the experimental participants was measured in the evaluation experiment in the walking environment shown in Fig. 13, with both the IMU and the OMC as the gold standard. An OMC with 24 cameras (VENUS3D, NOBBYTECH, Japan) and software for OMC (Motive: Tracker, NaturalPoint, Inc.) were used in the experimental environment as the reference system and as the gold standard, as indicated in Fig. 14. The marker for the OMC was attached to the center of the outer surface of the IMU. The OMC was calibrated such that the overall displacement error was less than 1 mm. The sampling frequency was set to 100 Hz for the OMC and IMU. After the measurement of both the IMU and OMC before the start of walking under the experimental conditions, the participants stomped their right and left feet once each to synchronize the IMU data and OMC data. Python 3.6 (Python Software Foundation) was used for data processing and analysis.

In addition, to validate the performance of the proposed method, the IMU measurement raw data were analyzed using not only the proposed method but also the representative previous method with a conventional typical SHS algorithm. This algorithm was based on a combination of simple integration and ZUPT. Specifically, simple double integration and ZUPT were used in acceleration related processes to estimate the local piecewise trajectory. For the turning angle, simple integration of the angular velocity was applied. The results of the trajectories analyzed by the conventional method were compared with OMC as well.

### Two evaluation experiments

A segmental evaluation was performed for the local stepwise trajectory of continuous gait trajectory, and an entire evaluation was conducted for the endpoint position of the overall continuous gait trajectory, which is the global destination of the continuous gait trajectory during walking.

### Segmental evaluation

In the segmental evaluation method, the stride length and turning angle for each single stride were evaluated using Pearson correlation analysis for the local piecewise trajectory of the continuous 3D foot trajectory. The stride length indicates the linear length from the foot ground contact to the next foot ground contact. The turning angle is the horizontal rotation angle of the walking direction from the foot ground contact to the next foot ground contact. Furthermore, the Bland–Altman analysis^24^ was introduced to evaluate the agreement between the IMU as the proposed method and the OMC as the gold standard. In the statistical analysis, the difference between the stride length and the turning angle between the IMU and the OMC was calculated as the estimation error of the proposed method. The turning angle in OMC was calculated using the angle output between adjacent stride vectors in the horizontal plane. The median and quartiles (1st quartile–3rd quartile) of the estimation errors were calculated from the results of the evaluation experiment.

### Entire evaluation

The normalized endpoint position error was evaluated throughout the evaluation, and it is the global destination of the continuous gait trajectory. In the statistical analysis, the normalized endpoint position error of the proposed method with the IMU was evaluated using the endpoint position of the measurement result of the OMC as the gold standard, which was normalized by the number of gait cycles. Further, the median and quartiles (1st quartile–3rd quartile) of the evaluation errors were calculated.
